# Flavonoids from *Selaginella doederleinii* Hieron and Their Antioxidant and Antiproliferative Activities

**DOI:** 10.3390/antiox11061189

**Published:** 2022-06-17

**Authors:** Felix Wambua Muema, Ye Liu, Yongli Zhang, Guilin Chen, Mingquan Guo

**Affiliations:** 1 CAS Key Laboratory of Plant Germplasm Enhancement and Specialty Agriculture, Wuhan Botanical Garden, Chinese Academy of Sciences, Wuhan 430074, China; fwambua83@mails.ucas.ac.cn (F.W.M.); liuye@wbgcas.cn (Y.L.); zhangyongli@wbgcas.cn (Y.Z.); glchen@wbgcas.cn (G.C.); 2 Sino-Africa Joint Research Center, Chinese Academy of Sciences, Wuhan 430074, China; 3 Innovation Academy for Drug Discovery and Development, Chinese Academy of Sciences, Shanghai 201203, China; 4 University of Chinese Academy of Sciences, Beijing 100049, China

**Keywords:** *Selaginella doederleinii* Hieron, antiproliferative, antioxidant, flavonoid, apigenin derivative

## Abstract

*Selaginella doederleinii* Hieron. (*S. doederleinii)* is a traditional herb that is widely used in China to treat several ailments, but mainly cancer. Studies have been carried out to determine the phytochemicals ascribed to its pharmacological activity. However, both phytochemical and pharmacological profiles have not been fully explored as few compounds have been reported. This study evaluated the flavonoid content of the ethanol extract and its four fractions (petroleum ether, dichloromethane, ethyl acetate, and *n*-butanol) together with their antioxidant activity (DPPH and FRAP assays). Further, the antiproliferative activity was evaluated. Two new secondary metabolites (**1** and **3**) were isolated from *S. doederleinii*, which comprised of an apigenin skeleton with a phenyl attached at C-8 of ring A and an acetyl group. Additionally, other known metabolites **2** and **4**–**16** were isolated, whereby compounds **2**, **4**, **5**, **8**, **12**, **15**, and **16** were reported for the first time in this species. These compounds were evaluated for their antioxidative potentials by both DPPH and FRAP assays, and for their antiproliferative activities by the MTT assay on three human cancer cell lines: colon cancer (HT-29), cervical cancer (HeLa), and lung cancer (A549). Compound **7** exhibited the best activity on the three cancer cell lines (HT-29, HeLa, A549) by inhibiting the rate of growth of the cancer cells in a dose-dependent manner with IC_50_ values of 27.97, 35.47, and 20.71 µM, respectively. The structure–activity relationship of the pure compounds was highlighted in this study. Hence, the study enriched both the phytochemical and pharmacological profiles of *S. doederleinii.*

## 1. Introduction

Cancer has persistently remained a global health concern by claiming many human lives [1]. In some developed countries, cancer incidences and the rate of mortality for many cancers have been reported to be decreasing. However, in developing countries, both morbidity and mortality rates are escalating at an alarming rate [2]. Screening and developing new anticancer chemotherapeutic drugs have remained an urgent approach in cancer management and mitigation [3]. Additionally, since a number of these phytochemicals’ solubility in water is poor, studies on the administration of plant extracts and pure isolated compounds to the delivery system are imperative. This would amplify their oral bioavailability and control the release of the drug payloads [4].

On the other hand, reactive oxygen species (ROS) are implicated in detrimental body health. They are chemical molecules that enclose oxygen in the form of superoxide hydroxyl radicals, peroxides, singlet oxygen, and hydrogen peroxide, which are generated by biological reactions in humans [5]. The production of ROS is usually in concentrations of picomolar and, when produced in excess amounts, they are neutralized in the body by the antioxidant system [6]. However, sometimes an imbalance between the ROS production and antioxidant protection system happens, hence there is oxidative stress [5], which potentially causes diseases such as diabetes and cancers [7,8]. To mitigate this, the intake of antioxidants that directly scavenge the free radicals or turn on molecules and enzymes that stimulate redox homeostasis can protect body cells from ROS-induced damage [9,10]. Natural antioxidants have increasingly attracted research focus as synthetic antioxidants have been associated with adverse effects on humans [11]. Additionally, anticancer agents derived from natural resources have been developed and approved to treat various types of cancer [12]. Therefore, phytochemicals are plant-based chemical constituents that occur naturally [13]. More than ten thousand phytochemicals have been identified and found to be of health benefits to humans in treating and reducing the risk of infection from several diseases [14,15]. Therefore, many researchers are focusing on naturally occurring phytochemicals for cancer treatment and prevention [3].

*Selaginella doederleinii* Hieron., commonly referred to as “da ye cai” and “shi shang bai” [16] in China, belongs to the genus *Selaginella* and family Selaginellaceae [17,18]. In China, the herb is distributed in the Guangxi Zhuang Autonomous Region and Yunnan and Guizhou provinces [19,20]. Traditionally, *S. doederleinii* has been used to treat cardiovascular disease [21], cancer [22,23], sore throat, and rheumatoid arthritis [24]. The decoction from this plant is normally prepared by boiling the whole plant in water. Owing to its traditional uses, studies evaluating its phytochemical and pharmacological properties have been carried out. The phytochemical studies of this species have revealed that it is composed of mainly biflavonoids [18,25], lignans [21], and alkaloids [26]; as well, Zou et al. [19] reported eight uncommon triflavonoids. Continued phytochemical research has led to the isolation of compounds with a unique apigenin skeleton structure and a phenyl attached at C-8 of ring A of the apigenin skeleton and flavonoids [27]. Pharmacological studies of *S. doederleinii* have revealed its antiproliferative [28,29,30], antioxidant [31,32], and anti-Alzheimer [19] activities. However, both phytochemical and pharmacological profiles of *S. doederleinii* have not been fully explored. More studies on the isolation and identification of novel compounds with significant biological activities are regarded as necessary as only a few compounds have been reported.

Therefore, this study aimed to explore the phytochemical constituents of *S. doederleinii* and evaluate its antioxidant and antiproliferative activities. To this end, the antioxidant potential of the ethanol extract, its fractions, and isolated compounds were evaluated by DPPH (2,2-diphenyl-1-picrylhydrazyl) and FRAP (ferric reducing antioxidant power) assays. The antiproliferative activities for both the ethanol extract, its fractions, and isolated compounds were evaluated by MTT (3-(4, 5-dimethylthiazol-2-yl)-2, 5-diphenyltetrazolium bromide) assay on three different human cancer cell lines: HT-29, Hela, and A549. Additionally, the study highlighted the structure–activity relationship of the evaluated compounds.

## 2. Materials and Methods

### 2.1. Plant Materials

*S. doederleinii* plant material was acquired from Bozhou Dianshitang Pharmaceutical Sales Co., Ltd., which were collected from Zhaotong City (China) and authenticated by Prof. Guangwan Hu from the Key Laboratory of Plant Germplasm Enhancement and Specialty Agriculture of Wuhan Botanical Garden, Chinese Academy of Sciences, Wuhan, China. A botanical specimen (20190710) was deposited at the herbarium of the institute.

### 2.2. Experimental Reagents and Instruments

Ethanol, petroleum ether, ethyl acetate, dichloromethane, and *n*-butanol were acquired from SinoPharm Chemical Reagent Co. Ltd. (Shanghai, China) and the HPLC-grade solvents (methanol, formic acid, and acetonitrile) were purchased from TEDIA Company Inc. (Fairfield, CA, USA). Chromatographic gels of ODS (YMC, Tokyo, Japan) and silica gel (Qingdao Marine Chemical Inc., Qingdao, China) were acquired. YMC-Pack ODS-A C18 (YMC, Tokyo, Japan) was used. 1,3,5-tri(2-pyridyl)-2,4,6-triazine (TPTZ), 2,2-diphenyl-1-picrylhydrazyl (DPPH), and vitamin C were purchased from Sigma-Aldrich Co. (St. Louis, MO, USA). Human cancer cell lines, human colon adenocarcinoma (HT-29), human cervical cancer (HeLa), and human lung adenocarcinoma (A549) were procured from American Type Culture Collection (ATCC, Manassas, VA, USA). HPLC Separation and purification were done with an Agilent 1100 series system with a YMC-Pack column (ODS-A, 250 × 10.0 mm I.D). NMR (1D and 2D) analysis were carried out on a Bruker-Avance-600 NMR spectrometer (Bruker, Karlsruhe, Germany). TMS was used as an internal standard. OD values were obtained on a Tecan Infinite M200 PRO multi-functional microplate reader (Männedorf, Switzerland). Ultra-pure water for HPLC was obtained from our laboratory using a Milli-Q system (Millipore, Billerica, MA, USA), Millipore membranes (0.22 µm).

### 2.3. Extraction and Separation

The dried plant materials (8.0 kg) were extracted by maceration with 75% ethanol (4 times, 3 days/time) at room temperature. The ethanol extract was evaporated under reduced pressure to obtain a residue (638.2 g). The obtained ethanol extract was then suspended in water for liquid–liquid extraction and successively extracted with petroleum ether (PE), dichloromethane (DCM), ethyl acetate (EA), and *n*-butanol (*n*-BuOH) to obtain their corresponding fractions.

The DCM extract (169.9 g) was subjected to an MCI gel column (MCI gel, 70–150 µm) to eliminate the dark color by eluting with MeOH-water at a ratio of 50:50, 80:20, and 100:0, and obtained four fractions (Fr. 1–4). Fr. 3 was separated by silica gel column chromatography (200–300 mesh) and passed with PE: DCM (2:1–1:3), 100% DCM, DCM: MeOH (20:1–1:1), and MeOH to obtain 13 fractions: A-M. Fr. G was further separated with MPLC (ODS C18, 5 µm) and eluted with MeOH-water in a ratio from 40:60 to 90:10 for 10.0 mL/min to give 12 subfractions: (Fr. G1-G12). Fr. F4 was separated on RP-HPLC (71% ACN-H_2_O, 2.5 mL/min, 280 nm) to obtain compound **14** (0.6 mg) and Fr. F5 (79% ACN-H_2_O, 2.5 mL/min, 280 nm) was separated to obtain compound **5** (0.7 mg). Fr. G4 was separated on RP-HPLC (77% MeOH-H_2_O, 2.5 mL/min, 280 nm) to obtain 9 peaks, which were further purified (68% ACN-H_2_O, 2.5 mL/min, 280 nm) to obtain compounds **3** (1.0 mg), **4** (1.0 mg), and **13** (1.4 mg). Fr. G6 was purified by RP-HPLC (72% ACN-H_2_O, 2.5 mL/min, 280 nm) to give compound **1** (1 mg), **8** (2.1 mg), and **12** (1 mg), and Fr. G7 (70% ACN-H_2_O, 2.5 mL/min, 280 nm) gave compounds **6** (1 mg), **15** (1.0 mg) and **16** (1.2 mg). Fr. G8 was purified by RP-HPLC (85% MeOH-H_2_O, 2.5 mL/min, 280 nm) to give compounds **7** (0.8 mg) and **9** (1.8 mg). Fr. G10 was purified (80% ACN-H_2_O, 2.5 mL/min, 280 nm) to obtain compound **11** (1.9 mg). Fr. H4 was separated (65–70%, MeOH-25 min, 3 mL/min), obtaining compounds **2** (0.8 mg) and **10** (0.5 mg).

### 2.4. Determination of the Total Flavonoid Content (TFC)

The TFC analysis was evaluated using the colorimetric method as described [33,34], with some modifications. Briefly, 80 µL of a diluted sample solution was mixed with NaNO_2_ (80 µL 5% *w*/*v*) solution and then shaken for 6 min. AlCl_3_ (80 µL 10% *w*/*v*) was added and allowed to stand for 6 min. Then, NaOH (400 µL 4% *w*/*v*) solution was added and allowed to react for 15 min. Afterward, the absorbance of the reaction mixture was read at 510 nm with a UV/VIS spectrophotometer (UV-11000, MAPADA, Shanghai, China) with methanol used as the blank. The TFC of each sample was evaluated in triplicate and expressed as rutin equivalents, which were determined from a rutin calibration curve (100–600 µg/mL), and the results were expressed as mg RE/g sample.

### 2.5. In Vitro Antioxidant Assays

#### 2.5.1. DPPH (2,2-diphenyl-1-picrylhydrazyl) Assay

The DPPH assay of *S. doederleinii* ethanol extract and four fractions was assessed as described in [35,36], with some minor modifications. Firstly, the DPPH solution was prepared with methanol at a concentration of 0.1 mM. Then, 10 µL of prepared samples and standards (vitamin C and BHT) of 9.375–250 µg/mL were added to 190 µL of the DPPH solution in each well of a 96-well plate. The mixture was shaken and incubated in darkness for 30 min. The absorbance of the reaction mixture was then taken at 517 nm using a multifunctional microplate reader (Tecan, Infinite M20PRO, Switzerland) with methanol being used as the blank. The analysis was done in triplicates and the results were expressed as the inhibition rate (%) and IC_50_ values. The DPPH radical scavenging activity was then calculated and expressed as follows:

DPPH radical scavenging activity (%) = [(A_0_ − A_1_/A_0_)] × 100% (1)

where A_0_ is the control absorbance and A_1_ is the sample/standard control absorbance.

#### 2.5.2. Ferric Reducing Antioxidant Power (FRAP) Assay

This assay was evaluated on the ethanol extract and its fractions (PE, DCM, EA, and *n*-BuOH) of *S. doederleinii* according to the reported method, with some slight modifications [37]. Firstly, a working solution, FRAP reagent comprised of 300 mM acetate buffer of pH 3.6, 20 mM FeCl_3_∙6H_2_O solution, and 10 mM TPTZ (2,4,6-tri(2-pyridyl)-S-triazine) solution in a ratio of 10:1:1 (*v*/*v*/*v*), was used. The working solution was then heated to 37 °C before use and 190 µL of FRAP working solution was mixed with 10 µL of sample in a 96-well plate. The mixture was then incubated at 37 °C for 10 min. The absorbance of the mixture was recorded by a microplate reader at a wavelength of 593 nm. The tests were done in triplicates and a standard curve was established. Eventually, the antioxidant activities were calculated and expressed as mmol Fe^2+^/g of the sample.

### 2.6. Antiproliferative Activity

The antiproliferative activity was performed by the MTT (3-(4, 5-dimethylthiazol-2-yl)-2, 5-diphenyltetrazolium bromide) method [38], with some modifications. Three human cancer cell lines, colon cancer (HT-29), cervical cancer (HeLa), and lung cancer (A549), were tested. The three cells were cultured in Dulbecco’s Modified Eagle’s Medium (DMEM), which was supplemented with 10% fetal bovine serum (FBS). The 90 µL cell suspension was added to each well and then the 96-well cell culture plates were maintained at 37 °C in a 5% CO_2_ atmosphere for 24 h to culture. Afterwards, 10 µL of samples at different concentrations (final concentration 6.25, 12.5, 25, 50, and 100 µM) were added to the wells in triplicates and the positive control was also set up. After incubating for 48 h, 15 µL of MTT (5 mg/mL) was added to each well and incubated at 37 °C for 4 h. Afterwards, 100 mL of DMSO was then added to each well and shaken for 15 min to dissolve the precipitates formed. The OD value of each well was measured at 590 nm with a microplate spectrophotometer reader (Tecan Infinite M200 PRO, TECAN, Männedorf, Switzerland). Then, the IC_50_ values were calculated by GraphPad Prism 8.0.1 Software (GraphPad Software Inc., San Diego, CA, USA).

### 2.7. Statistical Analysis

All the experiments were performed and data were expressed as mean ± standard deviation (SD) of triplicate values. Data analysis was performed by SPSS statistics 22 software (IBM Corporation, New York, NY, USA) using one-way ANOVA Duncan’s multiple range test and the significance difference was considered at *p* < 0.05. The IC_50_ values were calculated by GraphPad Prism 8.0 (GraphPad Software Inc, San Diego, CA, USA). Other software used in this study are: Chemoffice 18.0 (CambridgeSoft Corp, Cambridge, MA, USA), Origin 2019b (OriginLab Corporation, Northampton, MA, USA), and MestreNova (Mestrelab Research SL, San Diego, CA, USA).

## 3. Results and Discussion

### 3.1. Total Flavonoid Content

**Figure 1:**
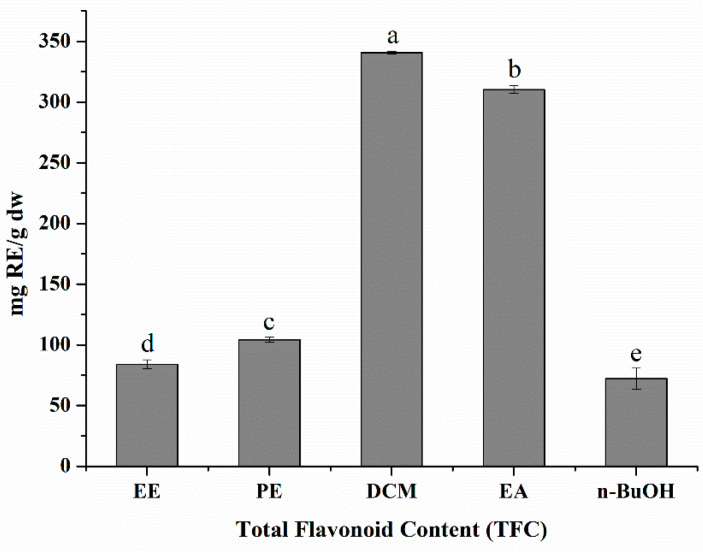
The total flavonoids content (TFC) of the ethanol extract and its fractions of *S. doederleinii* expressed rutin equivalents (RE) of dry weight sample. EE, ethanol extract; PE, petroleum ether; DCM, dichloromethane; EA, ethyl acetate; n-BuOH, n-butanol. All the data were expressed as mean ± standard deviation (*n* = 3). The letters ^(a–e)^ denote that the means are significantly different at a level of *p* < 0.05 (*n* = 3) by one-way ANOVA DMRT.

With *S. doederleinii* being used traditionally to treat cancer for decades and flavonoids having been shown as its main active constituents [25], it was necessary to evaluate its total flavonoid content (TFC). The flavonoid content was calculated using the equation, (y = 0.0013x + 0.0146, R^2^ = 0.9967), which was obtained by the calibration curve and ranged from 340.8 ± 1.0 to 72.2 ± 8.7 mg RE/g—with the dichloromethane fraction expressing the highest content and *n*-butanol the least, as shown in Figure 1; the order of the other constituents were: (2) ethyl acetate extract, (3) petroleum ether, and (4) crude extract with values of 310.3 ± 3.1, 104.2 ± 2.0, and 84.0 ± 3.6 mg RE/g, respectively. As seen from Figure 1, it could be noted that the TFC values of the DCM and EA fractions were close in range, with DCM being higher by 1.1 times. On the other hand, ethanolic extracts of *Selaginella tenera* and *Selaginella inaequalifolia* exhibited slightly higher TFC values of 125.6 ± 4.3 and 138.4 ± 2.1 mg RE/g, respectively [39], compared to our ethanol extract TFC content. The difference in values could be attributed to the extraction methodology and the geographical locations of the two species [40].

### 3.2. In-Vitro Antioxidant Potential of S. doederleinii Extracts

**Figure 2:**
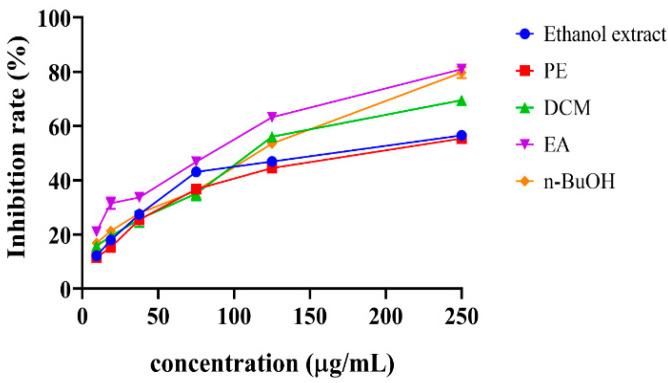
The radical scavenging percentage of ethanol extract and its PE (petroleum ether) fraction. DCM (dichloromethane), EA (ethyl acetate), and n-BuOH (n-butanol) of *S. doederleinii* by DPPH assay.

The ethanol extract and its fractions were evaluated for their scavenging potential and expressed different inhibition percentages. From Figure 2, the EA fraction expressed the highest inhibition percentage (DPPH) at a sample concentration of 250 µg/mL, followed by n-BuOH, DCM, ethanol extract, and PE with 80.9, 79.7, 69.5, 56.6, and 55.4%, respectively. The IC_50_ values for ethanol extract, its fractions, and positive controls were shown in Table 1. The EA fraction exhibited the best antioxidant activity, followed by DCM according to their IC_50_ values, while ethanol extract exhibited the lowest activity. However, the FRAP assay indicated that the DCM fraction had the highest reducing ability, followed by the EA fraction with 2.6 ± 0.1 and 1.7 ± 0.0 mmol Fe^2+/^g, respectively. Our fraction exhibited a slightly lower antioxidant activity compared to the DPPH assay results reported by Wang et al. [32]. In both assays, the ethanol extract and PE fraction exhibited the lowest scavenging activity, while both the DCM and EA fractions depicted strong activities, which were closely attributed with their TFC yields.

### 3.3. Antiproliferative Activity of S. doederleinii Extracts

**Figure 3:**
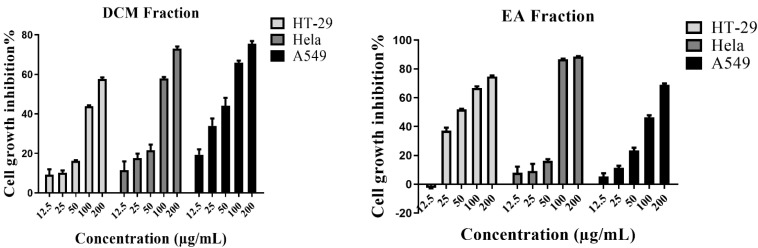
Antiproliferative activity in inhibition rate (%) at different concentrations of DCM (dichloromethane) and EA (ethyl acetate) fractions, respectively, against cancer cell lines HT-29, Hela, and A549 by the MTT assay. The cell growth inhibition rate of the ethanol extract, petroleum ether, and n-butanol could be expressed at a higher concentration than 200 µg/mL. The solvent’s inhibition rate was near zero value on all the cell lines. The data were expressed as mean ± SD (*n* = 3).

The antioxidant assays and TFC values revealed that both DCM and EA were the most active fractions of *S. doederleinii* when compared to the others. In this regard, both extracts were evaluated for their antiproliferative activity on three cancer cell lines: HT-29, HeLa, and A549 at different concentrations ranging from 12.5 to 200 µg/mL. The inhibition rates are shown in Figure 3, while the IC_50_ values are shown in Table 2. The inhibition rate of the solvent was almost zero, which confirmed that the solvent used did not influence the cytotoxicity of the samples. Additionally, the toxicity investigation revealed that the solvents did not influence cell viability. The EA fraction exhibited the best antiproliferative activity against the HT-29 and HeLa cell lines by inhibiting the cell growth rate in a dose-dependent manner with IC_50_ values of 55.6 ± 1.3 and 69.2 ± 1.3 µg/mL, respectively. The DCM fraction exhibited the best activity against the A549 cell line with an IC_50_ value of 55.9 ± 12.6 µg/mL. Song et al. [41] evaluated the anticancer activities of the extracts of *S. doederleinii* collected from different provinces in China against the A549 cancer cell line. Comparing the activities of the extracts with those of ours, our DCM extract exhibited better activity than most of the fractions. Our EA fraction exhibited better antiproliferative activity on the HeLa cancer cell line compared with that reported by Wang et al. [42], which had an IC_50_ value of 76.1 ± 1.9 µg/mL. These results explained the traditional use of *S. doederleinii* to cure and manage cancers. To this end, flavonoids expressed in the TFC results could be presumed to play a role in the antiproliferative activity of this species (both DCM and EA fractions) by suppressing the formation of cancers that emerge from oxidative stress. Accordingly, for a better understanding and exploration of this species towards cancer, the DCM fraction was selected for isolation work to identify the responsible bioactive phytochemicals.

### 3.4. Isolation and Structure Elucidation

**Figure 4:**
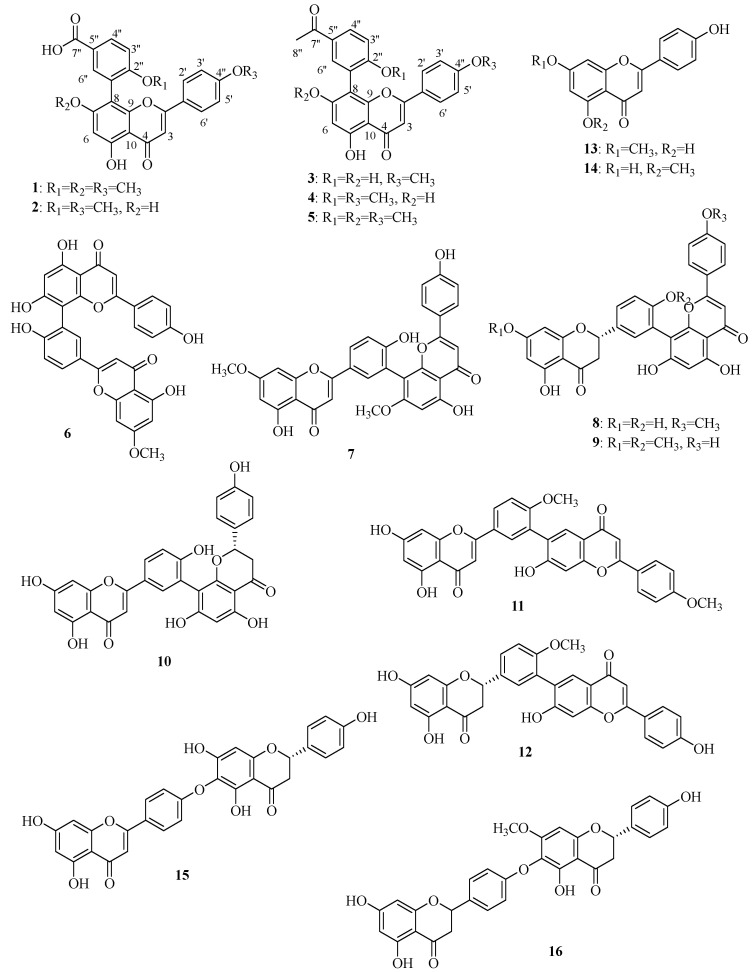
Chemical structures of isolated compounds (**1**–**16**) from DCM fraction of *S. doederleinii*.

A phytochemical examination of the DCM fraction of the whole plant of *S. doederleinii* using different column chromatography yielded two new compounds (**1** and **3**). Besides the new compounds, 14 other known compounds (Figure 4) were isolated and their chemical structures were determined by comparison of their NMR data (both ^1^H and ^13^C), as per existing literature.

**Figure 5:**
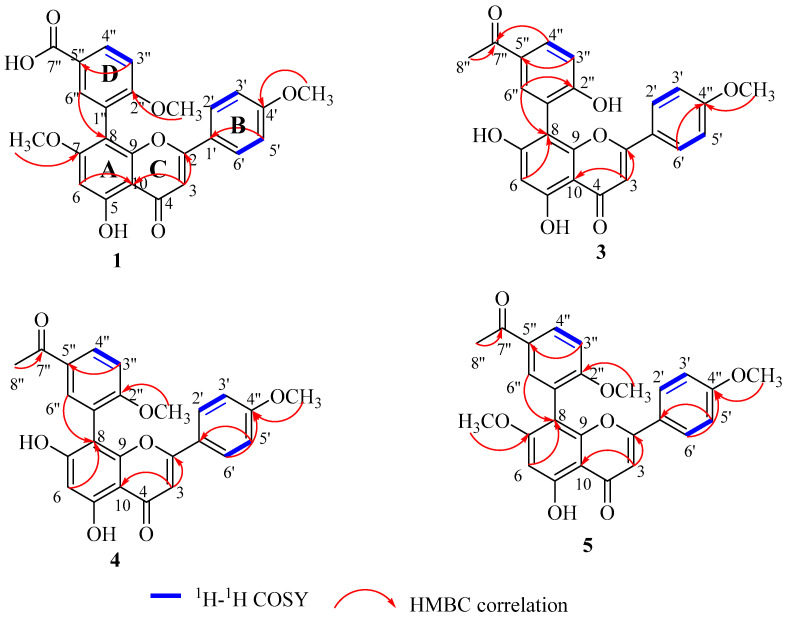
Main ^1^H-^1^H COSY and HMBC correlations of compounds **1**, **3**, **4** and **5**.

Compound **1** was isolated as a yellow amorphous powder. Its molecular formula was deduced as C_25_H_20_O_8,_ owing to a molecular ion peak observed at *m*/*z* 449.1227 [M + H]^+^ (calculated for 449.1231) in the HR-ESI-MS, as shown in Figure S2, which was per the ^1^H NMR and ^13^C NMR spectroscopic data (Table 3). Compound **1** consisted of a 1,2,5-trisubstituted benzene ring (ring D) at δ_H_ 8.13 (1H, dd, *J* = 8.7, 2.2 Hz, H-4″), 7.93 (1H, d, *J* = 2.2 Hz, H-6″), and δ_H_ 7.20 (1H, d, *J* = 8.7 Hz, H-3″). An AA′XX′ coupling system signal at δ_H_ 7.56 (2H, d, *J* = 8.9 Hz, H-2′, 6′) and δ_H_ 6.93 (2H, d, *J* = 8.9 Hz, H-3′, 5′) indicated the *para*-substitution of ring B. Two aromatic singlets were allocated to H-3 and H-6. The aromatic singlet at δ_H_ 6.68 was assigned to H-3 as it showed an HMBC correlation (Figure S8) with C-10 (δ_C_ 103.8) and C-2 (δ_C_ 164.6), and δ_H_ 6.60 was assigned to H-6 since H-8 was involved in the linkage between the flavonoid unit and the benzene ring (ring D). This was confirmed by the HMBC correlations from H-6″ (δ_H_ 7.93) to C-8 (δ_C_ 105.6), as shown in structure **1** in Figure 5. All 25 carbon resonances were resolved in the ^13^C NMR spectrum (Table 3 and Figure S4) and were further classified by a DEPT spectrum (Figure S5). They were categorized as 3 methyl (oxygenated), 9 methines (unsaturated), and 13 quaternary carbons (2 carbonyl).

The three methoxy groups were assigned to be attached to C-7, C-4′, and C-2″, which were determined by HMBC signals from δ_H_ 3.82 to δ_C_ 163.3 (C-7), δ_H_ 3.86 to δ_C_ 164.4 (C-4′), and δ_H_ 3.77 to δ_C_ 163.1 (C-2″). Besides the tri-substituted benzene ring (ring D), the remaining signals disclosed that Compound **1** had a flavonoid skeleton. The singlet proton at δ_H_ 6.60 (H-6) suggested that ring A could be substituted either at C-6 or C-8, and the HMBC studies have shown that the 1,2,5-trisubstituted benzene ring (ring D) was linked to C-8 by the correlation from δ_H_ 7.93 to δ_C_ 105.6 (C-8). It was concluded that compound **1** was an apigenin derivative and the chemical structure was determined as 3-(5-hydroxy-7-methoxy-2-(4-methoxyphenyl)-4-oxo-4H-chromen-8-yl)-4-methoxybenzoic acid.

Compound **3** was isolated as a yellow amorphous powder. Through HR-ESI-MS (positive ion mode) analysis, a molecular ion peak appeared at *m*/*z* 419.1121 [M + H]^+^ (calculated for [M+H]^+^ 419.1125) (Figure S10), indicating a molecular formula of C_24_H_18_O_7_ for **3**, which was per the ^1^H NMR and ^13^C NMR spectroscopic data (Table 3, Figures S11 and S12). The ^1^H NMR data of **3** displayed the presence of a 1,2,5-trisubstituted benzene moiety (ring D) at δ_H_ 8.01 (1H, dd, *J =* 8.4, 2.3 Hz, H-4″), 7.99 (1H, d, *J =* 2.2 Hz, H-6″), and 7.08 (1H, d, *J =* 8.5 Hz, H-3″), and was supported by the corresponding ^13^C NMR data (Table 3 and Figure S12). The singlet methyl group at δ_H_ 2.57 (3H, s, H-8″), along with the carbonyl carbon at δ_C_ 199.6, revealed an acetyl group that, based on the HMBC correlation of H-4″ and H-8″ to C-7″ (Figure 5), was attached to C-5″ of ring D. Additionally, an AA′XX′ coupling system signal at δ_H_ 7.64 (2H, d, *J =* 8.9 Hz, H-2′/6′) and 6.94 (2H, d, *J =* 8.9 Hz, H-3′/5′) indicated the *para*-substitution of ring B, and the two aromatic singlets at δ_H_ 6.68 and 6.40 were assigned to H-3 and H-6, respectively. All 24 carbons were displayed in the ^13^C NMR spectrum (Figure S12), which included 15 carbons for the apigenin skeleton, 6 for the phenyl (ring D), 2 for the acetyl group at δ_C_ 199.6 and 26.9, and 1 for the methoxyl at δ_C_ 56.0. The HMBC spectrum (Figure S16) displayed the presence of a C-1″-C-8 linkage in ring D and A by correlations from H-6″ (δ_H_ 7.99) to C-8 (δ_C_ 105.4), which confirmed that C-8 was the point of attachment of ring D to the apigenin skeleton. A methoxyl group was attached at C-4′ (δ_C_ 164.3) in ring B of the apigenin skeleton as it displayed HMBC correlations from δ_H_ 3.84 (OMe) to C-4′ (δ_C_ 164.3). At C-2″ in phenyl (ring D), a hydroxyl was attached as shown by HMBC correlations from H-4″/6″ (δ_H_ 8.01, 7.99) to C-2″ (δ_C_ 163.3), as shown in Figure 5 as well as the downfield shift resonance of C-2″ (δ_C_ 163.3) by 31.0 ppm. Hence, the structure of compound **3** was characterized as 8-(5-acetyl-2-hydroxyphenyl)-5,7-dihydroxy-2-(4-methoxyphenyl)-4H-chromen-4-one.

Compound **4** was obtained as a yellow amorphous powder and HR-ESI-MS exhibited a molecular peak at *m*/*z* 433.1276 [M + H]^+^ (calculated for 433.1282) with the molecular formula C_25_H_20_O_7_, which corresponded to the ^1^H NMR and ^13^C NMR spectroscopic data (Table 3). By comparing the ^1^H NMR and ^13^C NMR data of compound **3** to that of **4**, it was observed that there was an additional methoxyl that was attached at C-2″ of ring D, according to the HMBC correlations from δ_H_ 3.82 to C-2″ (δ_C_ 163.3). The structure of compound **4** was thus determined as 8-(5-acetyl-2-methoxyphenyl)-5,7-dihydroxy-2-(4-methoxyphenyl)-4H-chromen-4-one [43]. This is the first time its spectroscopic data and its isolation from natural resources have been reported.

Compound **5** was isolated as a yellow powder. It shared the same skeleton structure with compounds **3** and **4** but with three methoxy groups. ^1^H NMR and ^13^C NMR data gave the molecular formula as C_26_H_22_O_7_. The ^1^H NMR and ^13^C NMR spectroscopic data (Table 3) of **5** closely resembled that of **4,** except that the hydroxyl at C-7 of ring A was substituted by a methoxy group. The HMBC studies of this compound indicated that the three methoxy groups are attached at C-7, C-4′, and C-2″, as indicated in Figure 5. Hence, compound **5** was determined as 8-(5-acetyl-2-methoxyphenyl)-5-hydroxy-7-methoxy-2-(4-methoxyphenyl)-4H-chromen-4-one [43]. The spectroscopic data of **5** is also being reported for the first time in this study, as well as its isolation from natural resources.

The structures of 12 other known compounds, **2** and **6**–**16,** were established by comparison of their spectroscopic data with those reported in the literature as 3-(5,7-dihydroxy-2-(4-methoxy-phenyl)-4-oxo-4H-chromen-8-yl)-4-methoxy-benzoic acid (**2**) [44], Sequoiaflavone (**6**) [45], 7,7″-dimethyl ether amentoflavone (**7**) [22], 2,3-dihydro-4′′′-methyl ether amentoflavone (**8**) [46], 2,3-dihydro-7,4′-dimethyl ether amentoflavone (**9**) [47], 2″,3″-Dihydroamentoflavone (**10**) [48], 4′,4′′′-dimethyl ether robustaflavone (**11**) [47], 2,3-dihydro-4′-methyl ether robustaflavone (**12**) [49], 5,4′-dihydroxy-7-methoxyflavone (**13**) [50], thevetiaflavone (**14**) [51,52], 2″,3″-dihydrohinokiflavone (**15**) [53], and 7″-methyl ether tetrahydrohinokiflavone (**16**) [54].

Biflavonoids are the most common and characteristic compounds of the species *S. doederleinii,* with a few alkaloids [26], lignan [21], and triflavonoids [19] having been reported. In this study, we isolated five compounds with an apigenin skeleton and a phenyl (ring D) attached at C-8 of the apigenin and an acetyl attached at C-5″ of ring D. In genus *Selaginella*, many biflavonoids with a C-C interflavonoid connection at C-8 of apigenin have been reported [55]. By keenly observing compounds **1**–**5,** they resemble an amentoflavone (having C3′-C8″ interflavonoid linkage) derivative without the chromone part of the flavonoid I unit. Therefore, these five compounds could have been derived from these kinds of biflavonoids. Compounds with this kind of structure were first reported by Zou et al. [27] in this species. Four biflavonoids (**8**, **12**, **15**, and **16**) are being reported for the first time in this species. Additionally, compounds **2**, **13**, and **14** are reported for the first time in this species too.

### 3.5. Antioxidant Activities of Isolated Compounds from S. doederleinii

**Figure 6:**
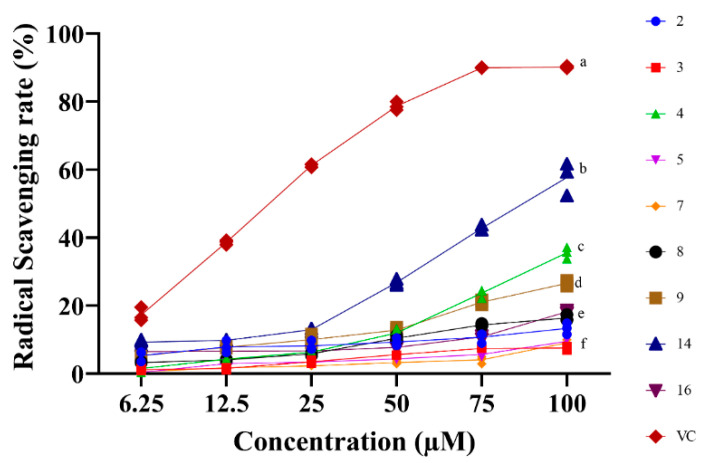
Free radical scavenging rates of isolated compounds from *S. doederleinii* by DPPH assay. The positive control used was Vitamin C (VC). Letters ^(a–f)^ indicate that the values are significantly different at a level of *p* < 0.05 (*n* = 3) by one-way ANOVA DMRT.

**Figure 7:**
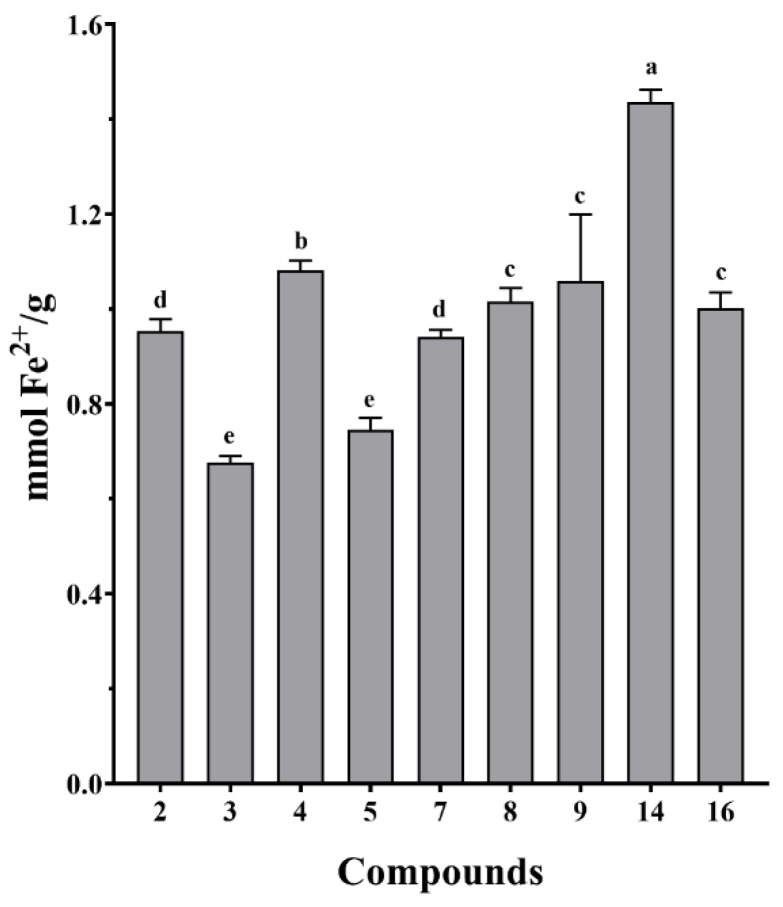
Antioxidant activities of isolated compounds from *S. doederleinii* evaluated by FRAP assay. The data were expressed as means ± SD (*n* = 3). The mean values denoted with letters ^(a–e)^ are significantly different at a level of *p* < 0.05 (*n* = 3) by one-way ANOVA DMRT.

The isolated compounds from *S. doederleinii* were evaluated for their antioxidant activity by DPPH and FRAP assays. All the examined compounds exhibited radical scavenging abilities at different concentrations with the lowest and highest concentrations of 6.25 and 100 µM, respectively, as shown in Figure 6. Compound **14** expressed the best antioxidant activity among the tested compounds with an IC_50_ value of 89.3 ± 4.0 µM, while the positive control (Vitamin C) had an IC_50_ value of 20.3 ± 0.2 µM. The radical scavenging ability of the isolated compounds from *S. doederleinii* is attributed to the hydroxy groups in their structures, which donate a hydrogen atom to neutralize the free radicals, hence suppressing their oxidation potentials. The tested compounds expressed close free radical scavenging abilities even at the highest concentration of 100 µM, except compound **14** which had a higher value as compared to the rest. The FRAP assay results (Figure 7) also revealed that compound **14** exhibited the highest ferric reducing ability with a value of 1.4 ± 0.03 mM Fe^2+^/g, followed by compound **4** with a value of 1.1 ± 0.02 mmol Fe^2+/^g, which also exhibited the second highest DPPH radical scavenging rate at concentration of 100 µM. Vitamin C was used as the positive control on the FRAP assay and it exhibited an ion-reducing capacity with a value of 7.8 ± 1.2 mM Fe^2+^/g. The antioxidant activity of the isolated compounds from *S. doederleinii* has not been reported before, hence our work reports this for the first time. Flavonoids derived from plants have been reported to be strong antioxidants [56]. Bedir et al. [44] evaluated the antioxidant activity of flavonoids and four biflavonoids (Amentoflavone, Bilobetin, Ginkgetin, and Sciadopitysin). The flavonoids exhibited noble antioxidant activity. However, none of the four biflavonoids evaluated exhibited strong antioxidant activity. Another study by Orčić et al. [57] revealed low antioxidant activity of biflavonoids isolated from *Hypericum perforatum* species, whereas the monomer flavonoids exhibited strong antioxidant activities. Previous studies in the same species had reported low antioxidant activities of isolated biflavonoids. This is in support of our findings, whereby flavonoid compound **14** exhibited the strongest antioxidant activity compared to the rest of the tested compounds, which were mainly biflavonoids.

### 3.6. Antiproliferation Activity of Compounds Isolated from S. doederleinii

All the isolated compounds were evaluated for their antiproliferation activity against three human cancer cell lines: HT-29, HeLa, and A549 by the MTT method. All the compounds showed antiproliferation activity on the three tested cancer cell lines to different degrees. Interestingly, these compounds expressed some level of antiproliferation on cancer cell line A549, which could give an insight into its major traditional use for lung cancer treatment and management. Among the 16 compounds, three (**8**, **9**, **16**) expressed the best activity by inhibiting the rate of cell growth in a dose-dependent manner on the three cancer cell lines, and their IC_50_ values were shown in Table 4. Interestingly, the three were biflavonoids, which have continued to be of interest in the search for cancer drugs [28,58]. Compounds **8** and **16** exhibited noble activities on cancer cell line A549 as compared to their activities on the other cell lines. This affirmed the DCM fraction antiproliferative activity on cancer cell line A549, which exhibited the best activity compared to the other cell lines. Additionally, it supports the main use of this species, which is traditionally in the treatment and management of lung cancer.

### 3.7. Structure–Activity Relationship of S. doederleinii Phytochemicals

The structure–activity relationship study of our results was interesting, with all compounds exhibiting obvious cytotoxicity on the three cancer cell lines. The two amentoflavone derivatives (**8** and **9**) exhibited antiproliferation activity, with **8** having the best activity on the HeLa and A549 cancer cell lines and **9** having the best activity on the HT-29 cell line, as shown in Table 4. Compound **8** exhibited better activity than **9**, this could be attributed to the OH at C-5,7 of ring A and C-4′ of ring B of the first flavonoid unit as compared to **9,** which had OCH_3_ at C-7,4′. This confirms the importance of OH at C-5,7 of ring A and at C-3′,4′ of ring B [59,60,61]. When comparing the antiproliferation of the hinokiflavone derivatives, compound **16** exhibited more interesting activity than **15** with the best activity on the three human cancer cell lines among the tested compounds. The noble activity of **16** was enhanced by the methoxy group at C-7″ of ring A of the second flavonoid unit. This was in accordance with Du et al. [62], who established that methylation at ring A enhances the antiproliferative activity of flavones.

## 4. Conclusions

In this study, the TFC, antioxidant (DPPH and FRAP assays), and antiproliferative potentials of the ethanol extract and its fractions were evaluated. The DCM and EA fractions depicted good potency on the three bioassays. The phytochemical investigation was carried out to identify the phytochemicals responsible for its antioxidant and antiproliferative activities. This resulted in the isolation of 16 compounds, including two new compounds (**1** and **3**). The isolated compounds were then evaluated for their antioxidative and antiproliferative potentials. All the evaluated compounds exhibited some free radical scavenging ability. Compound **14** expressed the best antioxidant activity on the DPPH assay and the highest ferric reducing antioxidant ability on the FRAP assay. The antiproliferative activity was tested by MTT assay on three human cancer cell lines: HT-29, HeLa, and A549. Compound 16 (7″-methyl ether Tetrahydrohinokiflavone) exhibited the strongest activity by inhibiting the rate of cell growth in a dose-dependent manner on the three cancer cell lines. Compounds **8** and **16** exhibited noble antiproliferative activities on the A549 cancer cell line, hence they could be promising lung cancer drug candidates. The study has therefore supported the traditional use of *S. doederleinii* in cancer treatment and identified the bioactive chemical constituents responsible for its pharmacological properties. Additionally, the study has enriched the phytochemical constitution of *S. doederleinii* as well as its pharmacological profile. However, we strongly suggest more isolation work to expand the phytochemical profile of this species with new compounds of different classes as it has been reported in other species of the genus *Selaginella*.

## Figures and Tables

**Table 1:** Antioxidant activities of ethanol extract of *S. doederleinii* and its PE, DCM, EA, and n-BuOH fractions, and positive controls of Vitamin C and BHT evaluated by DPPH and FRAP assays.

Sample	DPPH	FRAP
	**IC_50_ (µg/mL)**	**mmol Fe^2+^/g**
Ethanol extract	187.5 ± 1.3 ^a^	1.1 ± 0.0 ^c^
PE	176.5 ± 0.8 ^b^	0.9 ± 0.1 ^c^
DCM	110.6 ± 1.3 ^d^	2.6 ± 0.1 ^b^
EA	82.1 ± 1.1 ^e^	1.7 ± 0.0 ^bc^
n-BuOH	115.2 ± 1.4 ^c^	1.6 ± 0.0 ^bc^
Vitamin C	5.8 ± 1.9 ^f^	7.8 ± 1.2 ^a^
BHT	5.9 ± 1.6 ^f^	Nt

Data were expressed as means ± standard deviation (*n* = 3). The mean values denoted by letters ^(a–f)^ are significantly different at level *p* < 0.05 by one-way ANOVA DMRT. Nt denote, not tested.

**Table 2:** IC_50_ values of DCM and EA fractions on HT-29, HeLa, and A549.

Fraction	IC_50_ (µg/mL)		
	HT-29	HeLa	A549
**DCM**	145.4 ± 3.0	92.5 ± 0.6	55.9 ± 12.6
**EA**	55.6 ± 1.3	69.2 ± 1.3	112.7 ± 6.7

Data expressed as means ± standard deviation (*n* = 3).

**Table 3:** ^1^H NMR and ^13^C NMR data of compounds **1**, **3**–**5**.

	1		3		4		5	
H/C	δ_H_ (*J* in Hz)	δ_C_	δ_H_ (*J* in Hz)	δ_C_	δ_H_ (*J* in Hz)	δ_C_	δ_H_ (*J* in Hz)	δ_C_
2		164.6		165.7		163.5		163.4
3	6.68 1H, s	103.8	6.68 1H, s	103.9	6.67 1H, s	103.9	6.71 1H, s	103.9
4		184.5		184.3		179.4		179.4
5		163.1		162.5				164.6
6	6.60 1H, s	96.4	6.40 1H, s	100.1	6.38 1H, s	99.9	6.63 1H, s	96.4
7		163.3		166.7		164.4		164.4
8		105.6		105.4		104.6		105.7
9		155.5		156.5		156.8		155.5
10		103.8		104.3		104.9		106.4
1′		124.3		124.6		124.7		124.3
2′/6′	7.56 2H, d, *J* = 8.9 Hz	129.1	7.64 2H, d, *J* = 8.9 Hz	129.1	7.54 2H, d, *J* = 8.9 Hz	129.0	7.56 2H, d, *J* = 8.9 Hz	129.0
3′/5′	6.93 2H, d, *J* = 8.9 Hz	115.5	6.94 2H, d, *J* = 8.9 Hz	115.5	6.93 2H, d, *J* = 8.9 Hz	115.5	6.94 2H, d, *J* = 8.9 Hz	115.5
4′		164.4		164.3		164.4		165.0
1″		122.0		119.7		122.6		122.5
2″		163.1		163.3		163.3		163.3
3″	7.20 1H, d, *J* = 8.7 Hz	111.4	7.08 1H, d, *J* = 8.5 Hz	116.7	7.24 1H, d, *J* = 8.8 Hz	111.8	7.28 1H, d, *J* = 8.8 Hz	111.7
4″	8.13 1H, dd, *J* = 8.7, 2.2 Hz	132.7	8.01 1H, dd, *J* = 8.4, 2.3 Hz	130.9	8.16 1H, dd, *J* = 8.7, 2.4 Hz	131.9	8.17 1H, dd, *J* = 8.7, 2.3 Hz	131.1
5″		126.2		130.1		130.9		130.9
6″	7.93 1H, d, *J* = 2.2 Hz	135.5	7.99 1H, d, *J* = 2.2 Hz	135.7	7.99 1H, d, *J* = 2.3 Hz	134.8	7.96 1H, d, *J* = 2.3 Hz	134.6
7″		165.9		199.6		196.5		199.5
8″			2.57 3H, s	26.9	2.57 3H, s	26.9	2.57 3H, s	28.1
OMe-7	3.82 3H, s	56.0					3.84 3H, s	56.9
OMe-4′	3.86 3H, s	56.8	3.84 3H, s	56.0	3.82 3H, s	56.5	3.87 3H, s	56.5
OMe-2″	3.77 3H, s	56.3			3.82 3H, s	56.0	3.82 3H, S	56.0

600 MHz ^1^H-NMR and 150 MHz ^13^C-NMR data recorded in Methanol-*d*_4._

**Table 4:** IC_50_ values of compounds **8**, **9**, and **16** on **HT-29**, **HeLa,** and **A549**.

Compound No.	IC50 (µM)		
	HT-29	HeLa	A549
**8**	56.9 ± 3.4	43.5 ± 4.2	24.3 ± 1.2
**9**	44.7 ± 3.2	>100	>100
**16**	27.9 ± 1.0	35.5 ± 4.2	20.7 ± 3.4

## Data Availability

Not applicable.

## Supplementary Materials

The following supporting information can be downloaded at: https://www.mdpi.com/article/10.3390/antiox11061189/s1. Figure S1. Isolation flow diagram of compounds **1–16**, Figure S2. UPLC-QTOF-MS spectrum of compound **1**, Figure S3. 1H NMR (600 MHz) spectrum of compound **1** in Methanol-*d*4, Figure S4. 13C NMR (150 MHz) spectrum of compound **1** in Methanol-*d*4, Figure S5. DEPT (150 MHz) spectrum of compound **1** in Methanol-*d*4, Figure S6. HSQC (600 MHz) spectrum of compound **1** in Methanol-*d*4, Figure S7. 1H-1H COSY (600 MHz) spectrum of compound **1** in Methanol-*d*4, Figure S8. HMBC (600 MHz) spectrum of compound **1** in Methanol-*d*4, Figure S9. NOESY (600 MHz) spectrum of compound **1** in Methanol-*d*4, Figure S10. UPLC-QTOF-MS spectrum of compound **3**, Figure S11. 1H NMR (600 MHz) spectrum of compound **3** in Methanol-*d*4, Figure S12. 13C NMR (150 MHz) spectrum of compound **3** in Methanol-*d*4, Figure S13. DEPT (150 MHz) spectrum of compound **3** in Methanol-*d*4, Figure S14. HSQC (600 MHz) spectrum of compound **3** in Methanol-*d*4, Figure S15. 1H-1H COSY (600 MHz) spectrum of compound **3** in Methanol-*d*4, Figure S16. HMBC (600 MHz) spectrum of compound **3** in Methanol-*d*4, Figure S17. NOESY (600 MHz) spectrum of compound **3** in Methanol-*d*4.
