# Supplementary material for: Flavonoids from Selaginella doederleinii Hieron and Their Antioxidant and Antiproliferative Activities
Source: Antioxidants (Basel). 2022 Jun 17;11(6):1189. doi: 10.3390/antiox11061189 (PMC9229023; doi:10.3390/antiox11061189)
Supplement: Supplementary file 1 [file antioxidants-11-01189-s001.zip › antioxidants-1750303-supplementary.pdf]

Supplementary data

Flavonoids from Selaginella doederleinii Hieron and Their Antioxidant and Antiproliferative Activities

Felix Wambua Muema[1,2,3,4,†], Ye Liu[1,2,3,†], Yongli Zhang[1,2,3], Guilin Chen[1,2,3] and Mingquan Guo[1,2,3,*]

1 CAS Key Laboratory of Plant Germplasm Enhancement and Specialty Agriculture, Wuhan Botanical Garden, Chinese Academy of Sciences, Wuhan 430074, China; fwambua83@mails.ucas.ac.cn (F.W.M.); liuye@wbgcas.cn (Y.L.); zhangyongli@wbgcas.cn (Y.Z.); glchen@wbgcas.cn.
2 Sino-Africa Joint Research Center, Chinese Academy of Sciences, Wuhan 430074, China.
3 Innovation Academy for Drug Discovery and Development, Chinese Academy of Sciences, Shanghai 201203, China.
4 University of Chinese Academy of Sciences, Beijing 100049, China.
* Correspondence: guomq@wbgcas.cn; Tel.: +86-027-87700850
† These authors contributed equally to this work

Supporting data content

Figure S1. Isolation flow diagram of compounds **1-16**

Figure S2. UPLC-QTOF-MS spectrum of compound **1**

Figure S3. ^{1}H NMR (600 MHz) spectrum of compound **1** in Methanol-*d*4

Figure S4. ^{13}C NMR (150 MHz) spectrum of compound **1** in Methanol-*d*4

Figure S5. DEPT (150 MHz) spectrum of compound **1** in Methanol-*d*4

Figure S6. HSQC (600 MHz) spectrum of compound **1** in Methanol-*d*4

Figure S7. ^{1}H-^{1}H COSY (600 MHz) spectrum of compound **1** in Methanol- *d*4

Figure S8. HMBC (600 MHz) spectrum of compound **1** in Methanol-*d*4

Figure S9. NOESY (600 MHz) spectrum of compound **1** in Methanol-*d*4

Figure S10. UPLC-QTOF-MS spectrum of compound **3**

Figure S11. ^{1}H NMR (600 MHz) spectrum of compound **3** in Methanol-*d*4

Figure S12. ^{13}C NMR (150 MHz) spectrum of compound **3** in Methanol-*d*4

Figure S13. DEPT (150 MHz) spectrum of compound **3** in Methanol-*d*4

Figure S14. HSQC (600 MHz) spectrum of compound **3** in Methanol-*d*4

Figure S15. ^{1}H-^{1}H COSY (600 MHz) spectrum of compound **3** in Methanol-*d*4

Figure S16. HMBC (600 MHz) spectrum of compound **3** in Methanol-*d*4

Figure S17. NOESY (600 MHz) spectrum of compound **3** in Methanol-*d*4

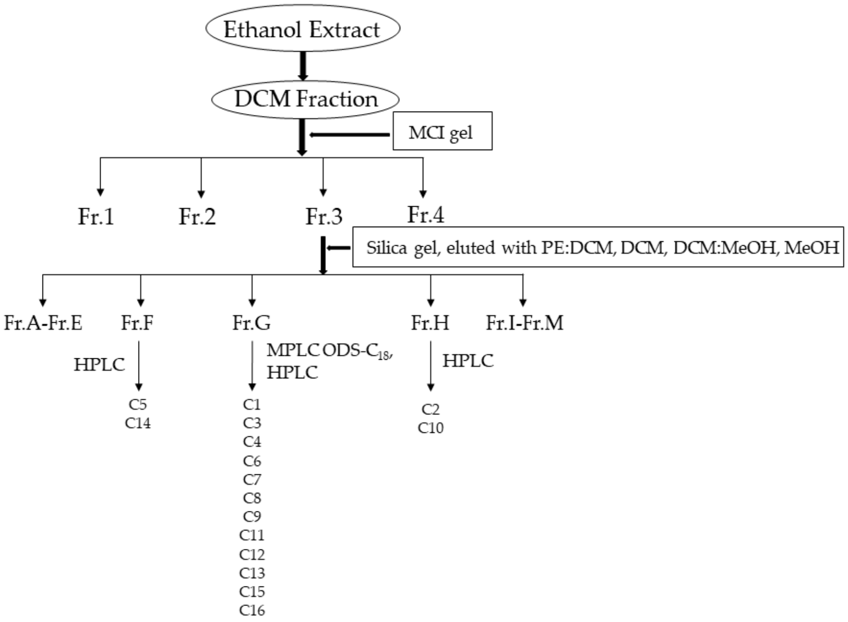

Figure S1. Isolation flow diagram of compounds **1-16**

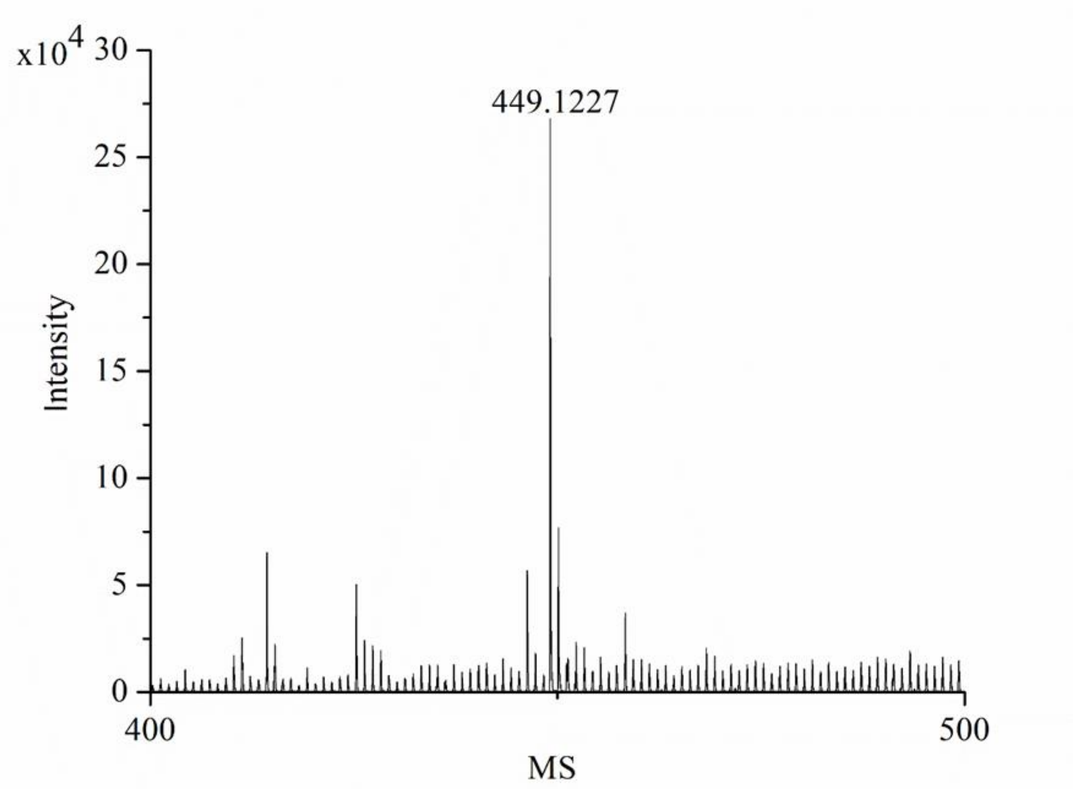

Figure S2. UPLC-QTOF-MS spectrum of compound **1**

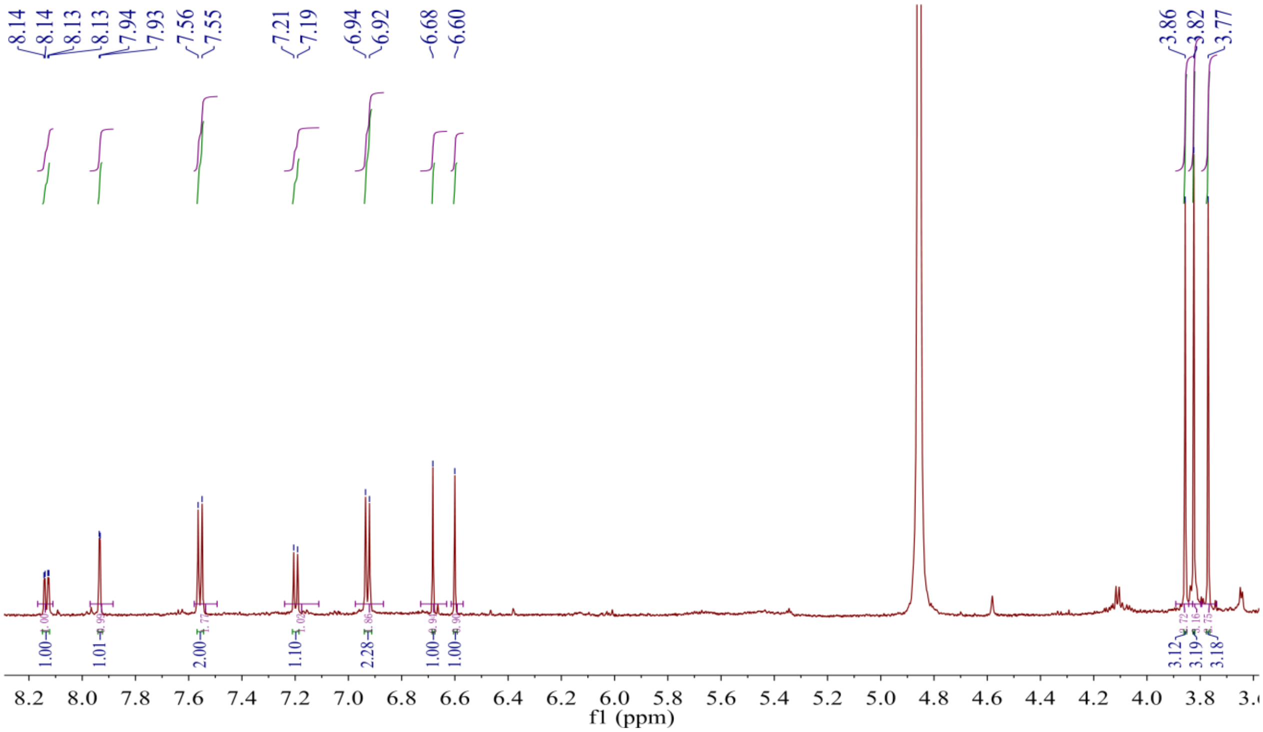

Figure S3. ^{1}H NMR (600 MHz) spectrum of compound **1** in Methanol-*d*4

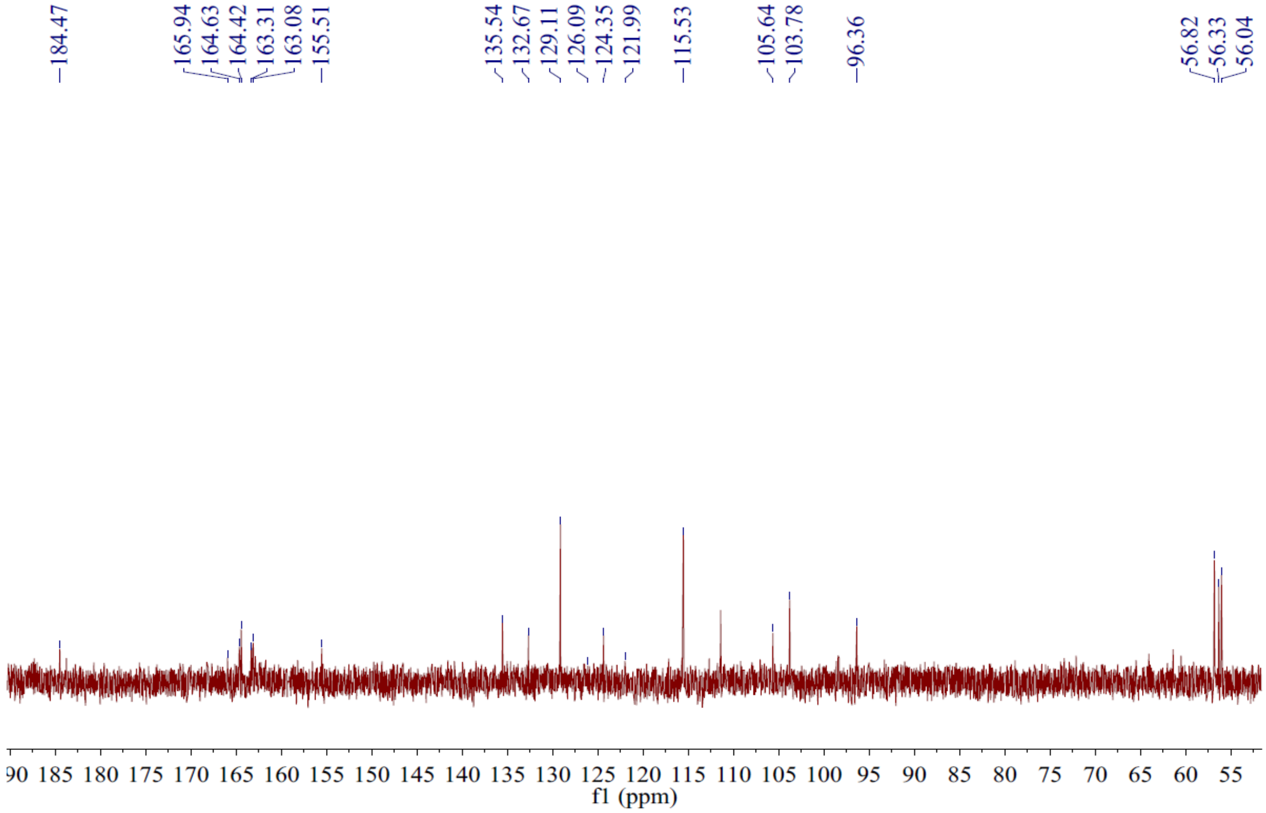

Figure S4. ^{13}C NMR (150 MHz) spectrum of compound **1** in Methanol-*d*4

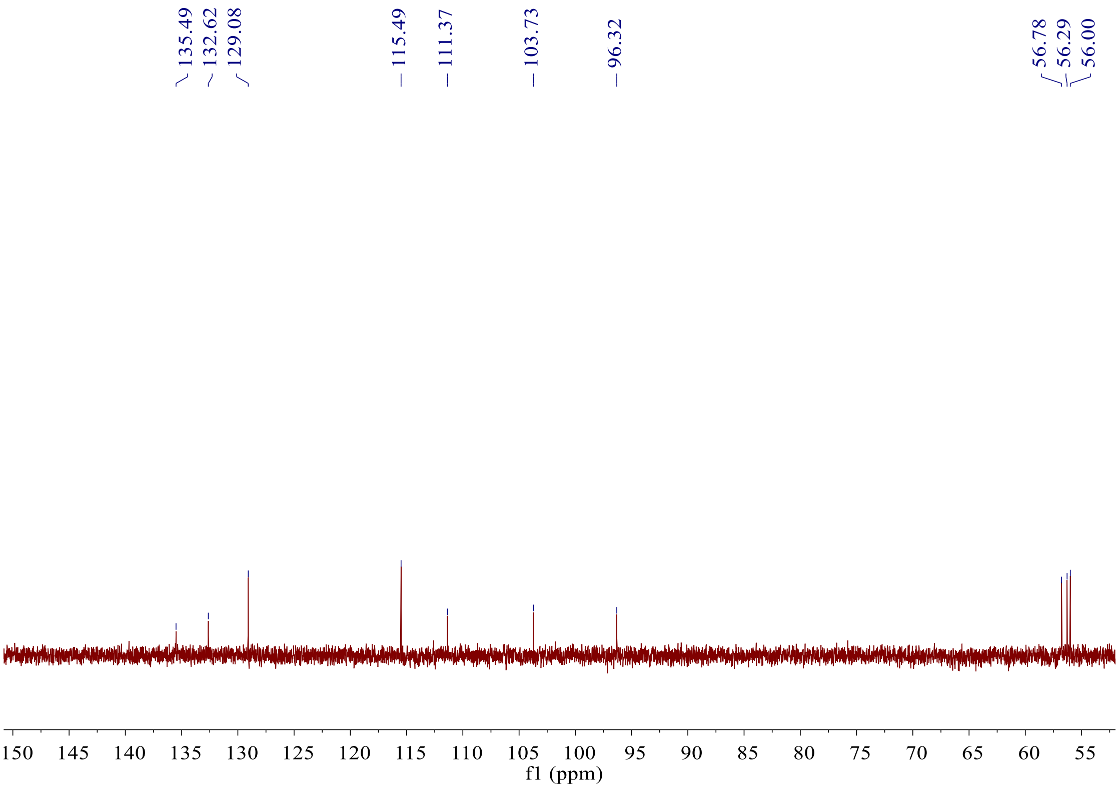

Figure S5. DEPT (150 MHz) spectrum of compound **1** in Methanol-*d*4

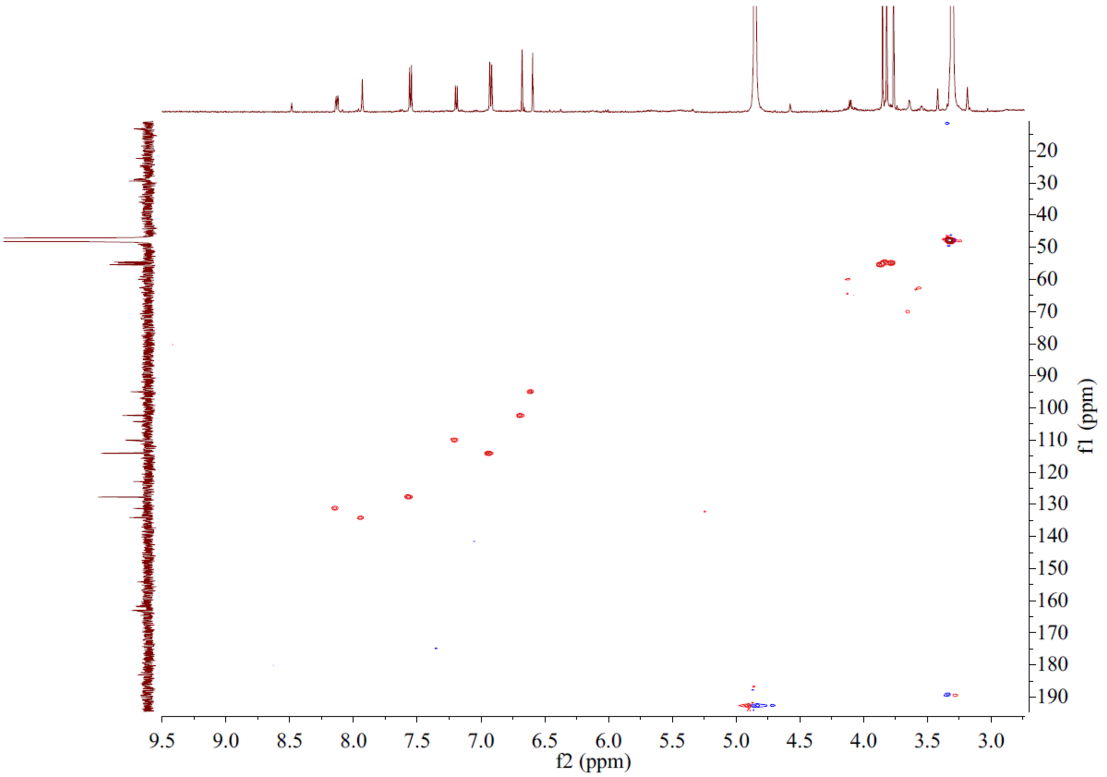

Figure S6. HSQC (600 MHz) spectrum of compound **1** in Methanol-*d*4

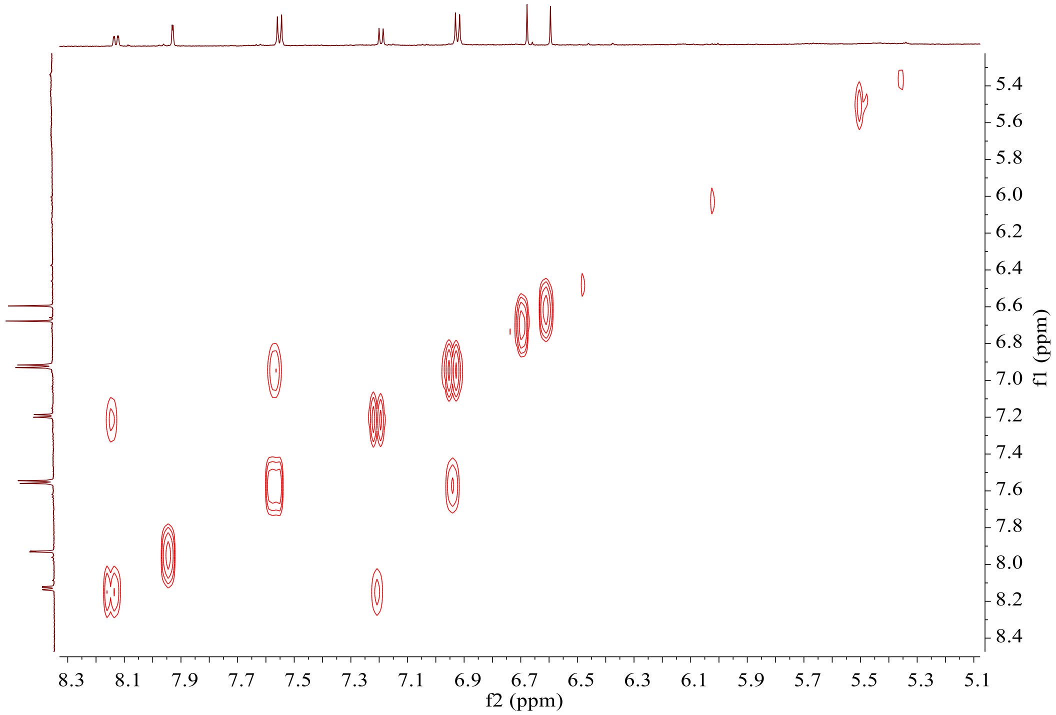

Figure S7. ^{1}H-^{1}H COSY (600 MHz) spectrum of compound **1** in Methanol-*d*4

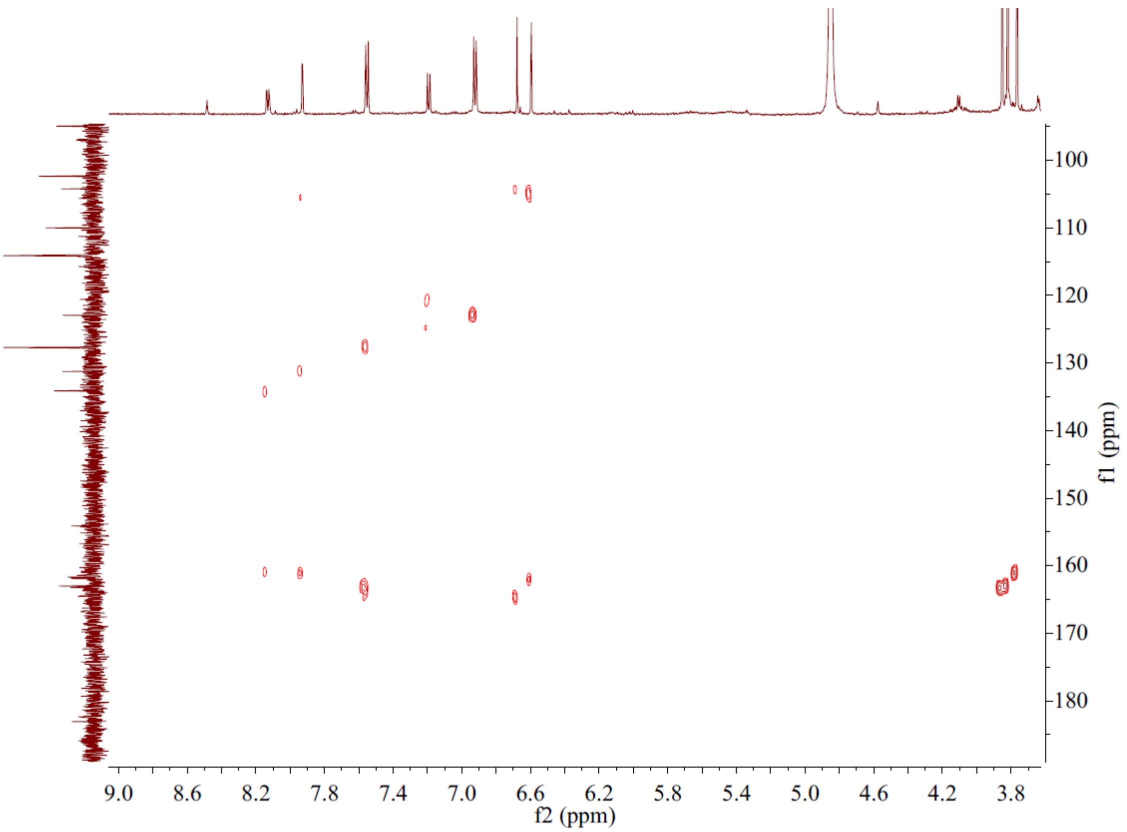

Figure S8. HMBC (600 MHz) spectrum of compound **1** in Methanol-*d*4

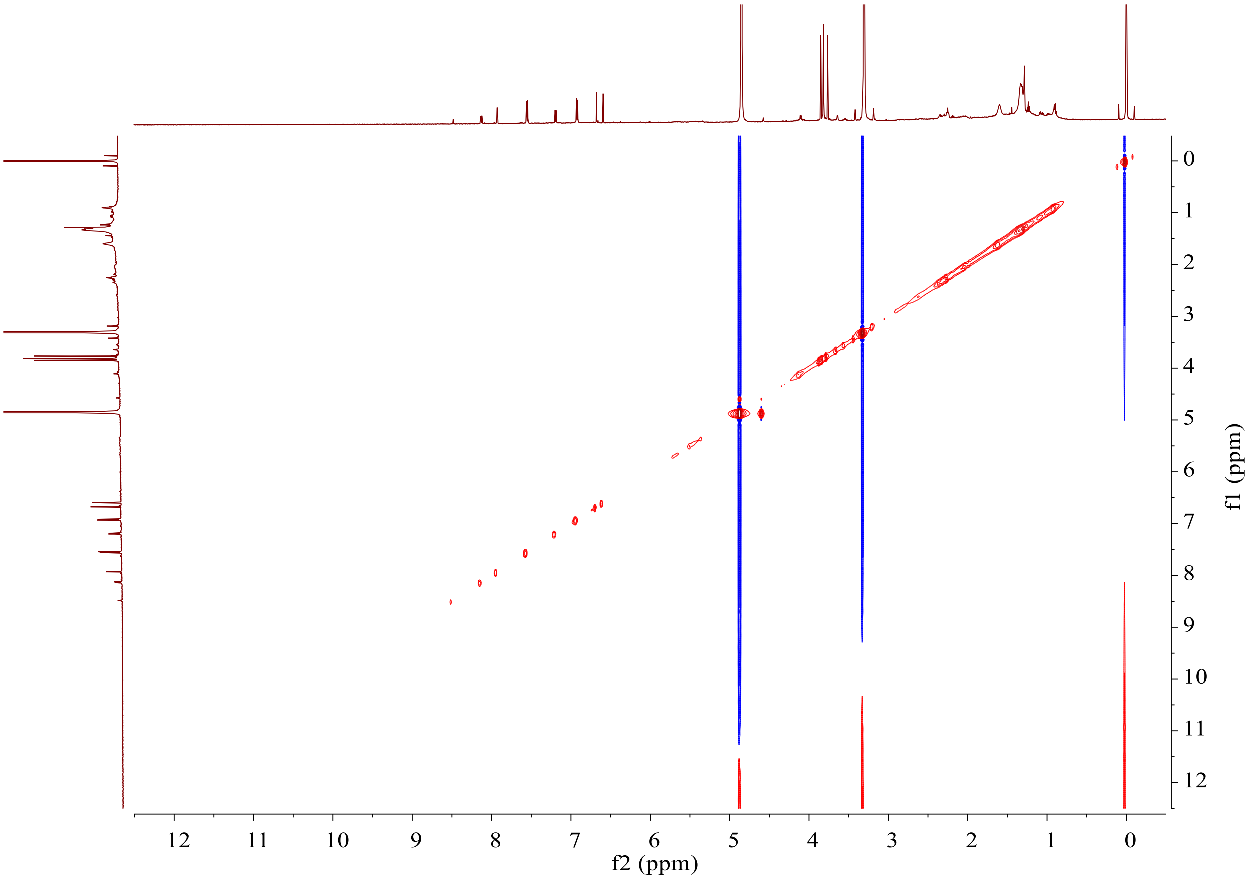

Figure S9. NOESY (600 MHz) spectrum of compound **1** in Methanol-*d*4

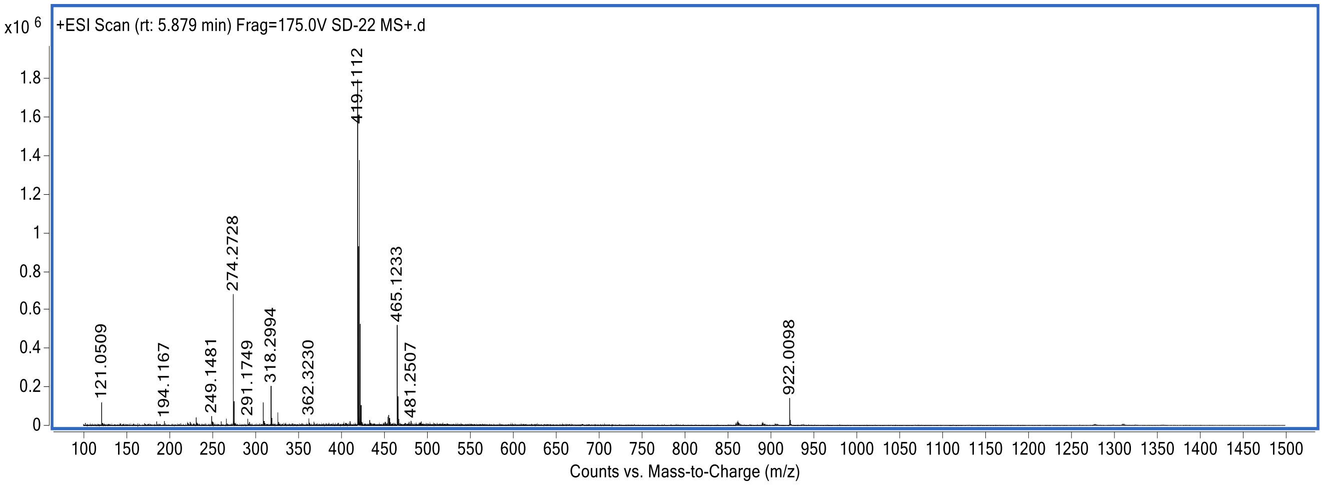

Figure S10. UPLC-QTOF-MS spectrum of compound **3**

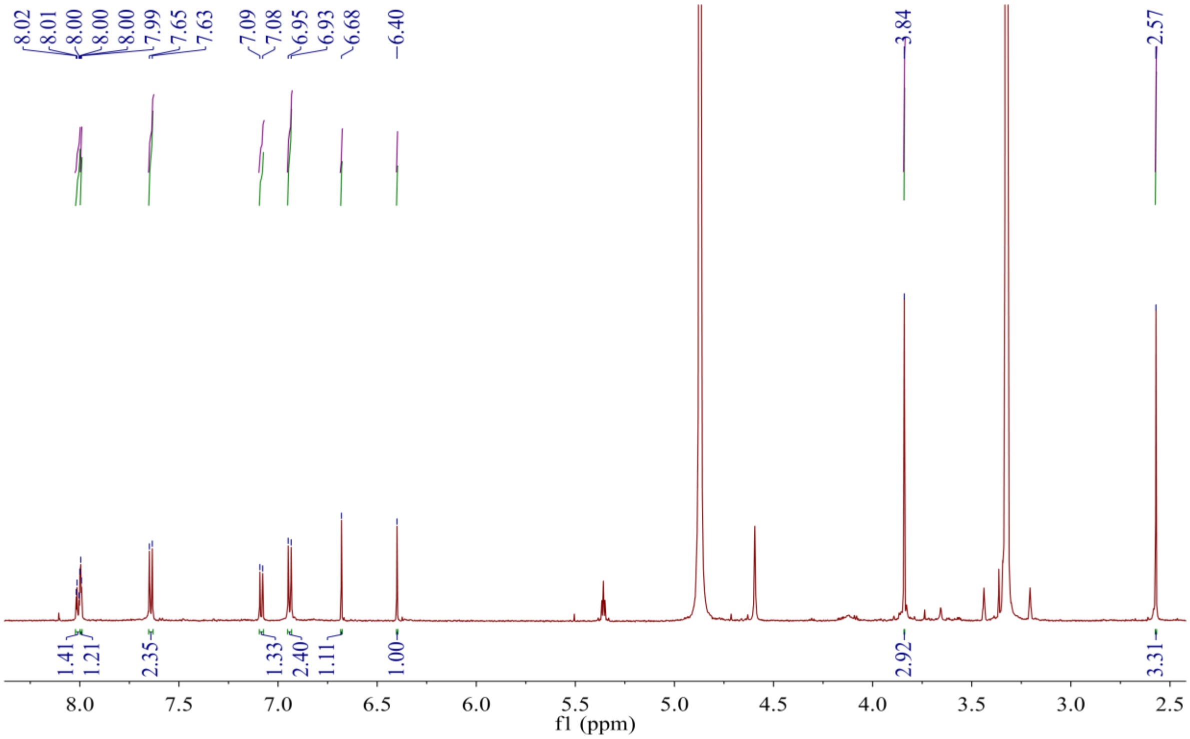

Figure S11. ^{1}H NMR (600 MHz) spectrum of compound **3** in Methanol-*d*4

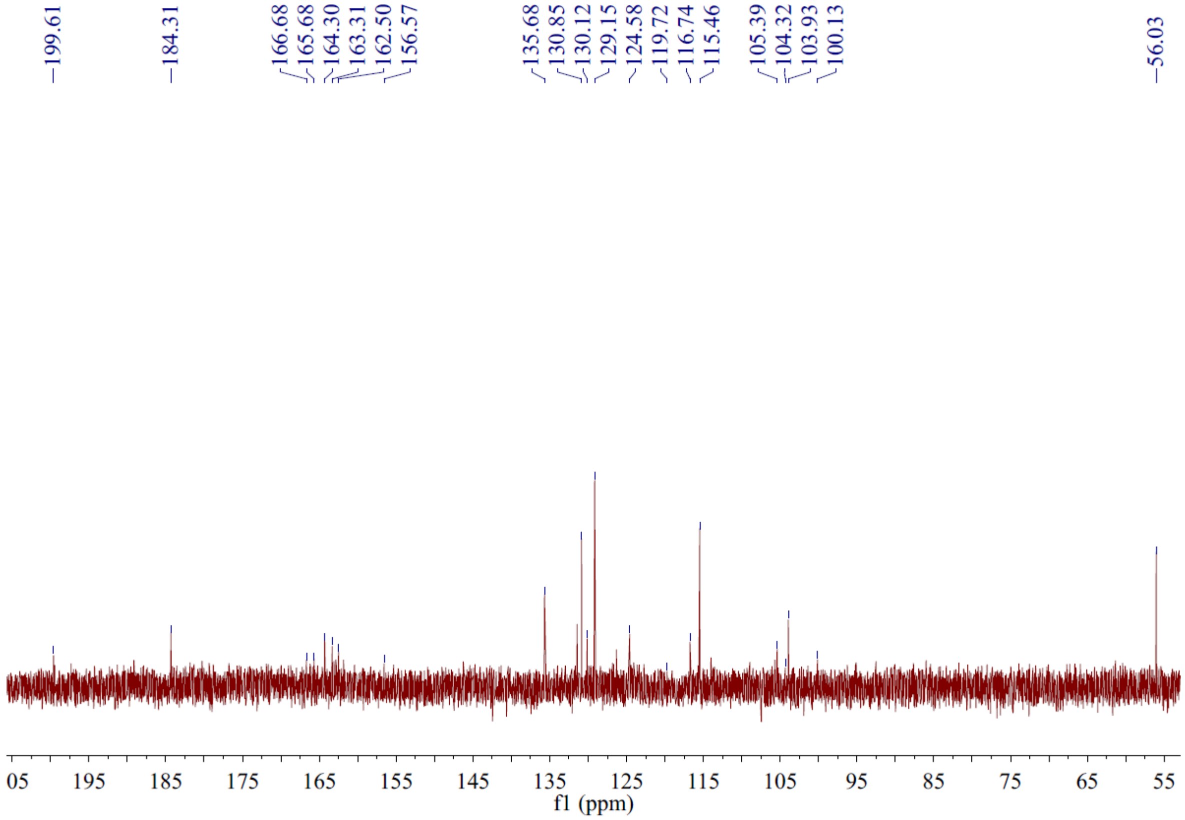

Figure S12. ^{13}C NMR (150 MHz) spectrum of compound **3** in Methanol-*d*4

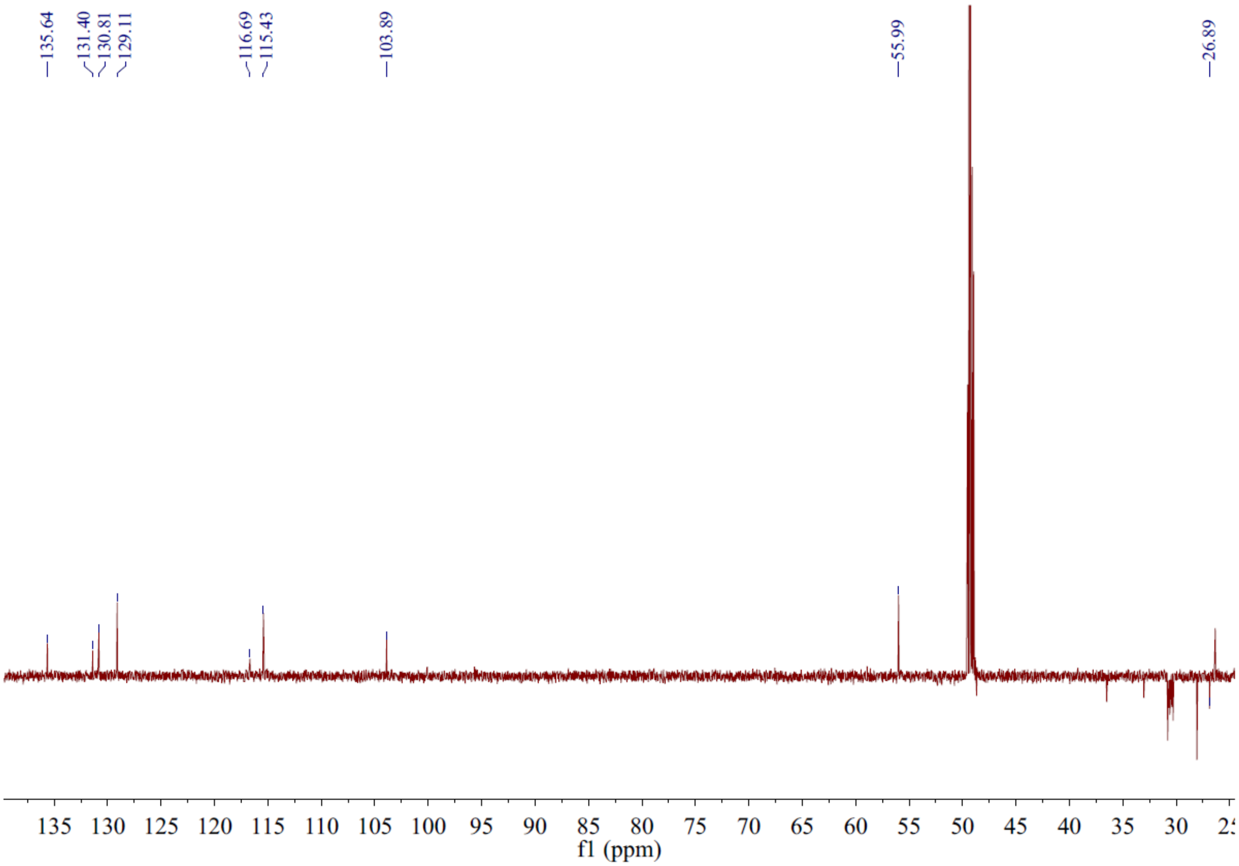

Figure S13. DEPT (150 MHz) spectrum of compound **3** in Methanol-*d*4

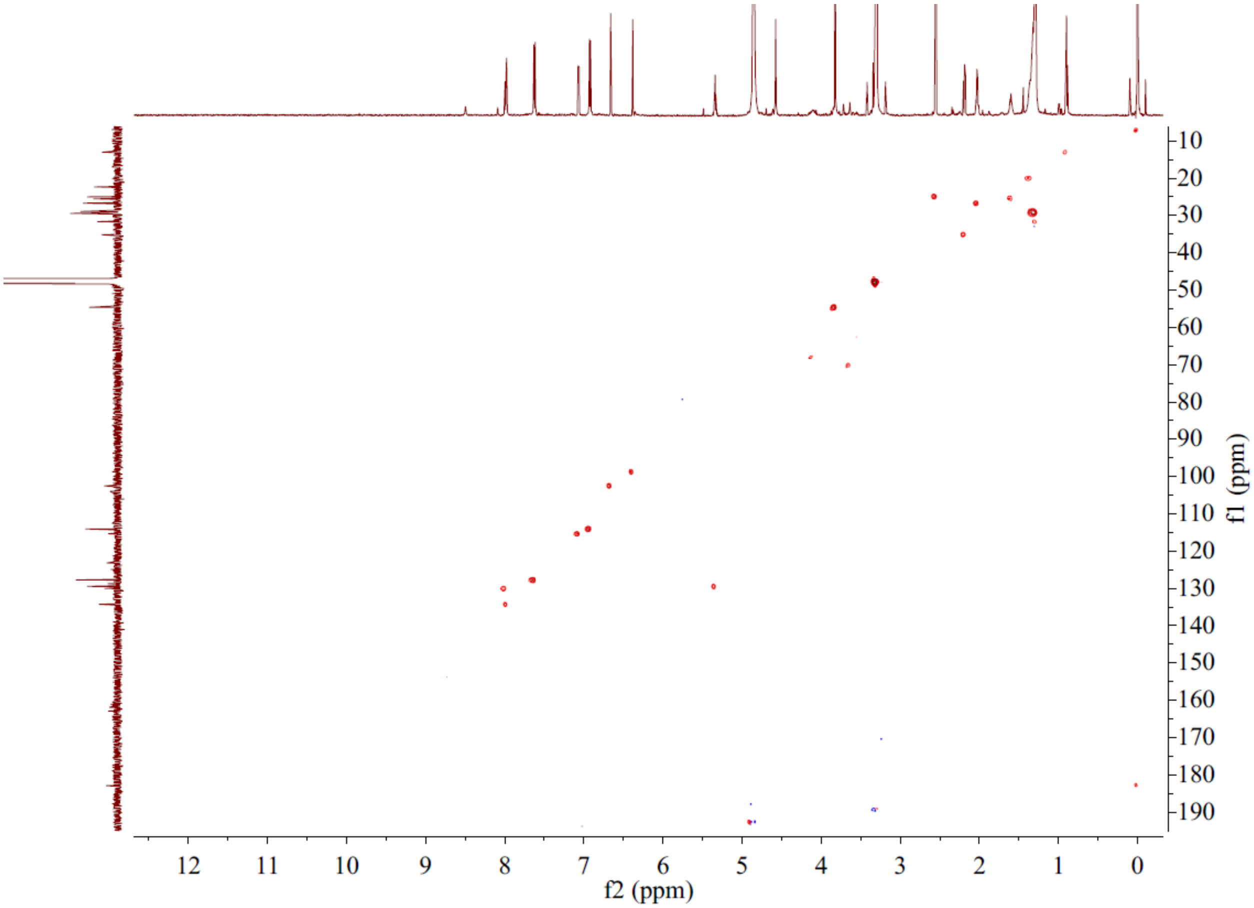

Figure S14. HSQC (600 MHz) spectrum of compound **3** in Methanol-*d*4

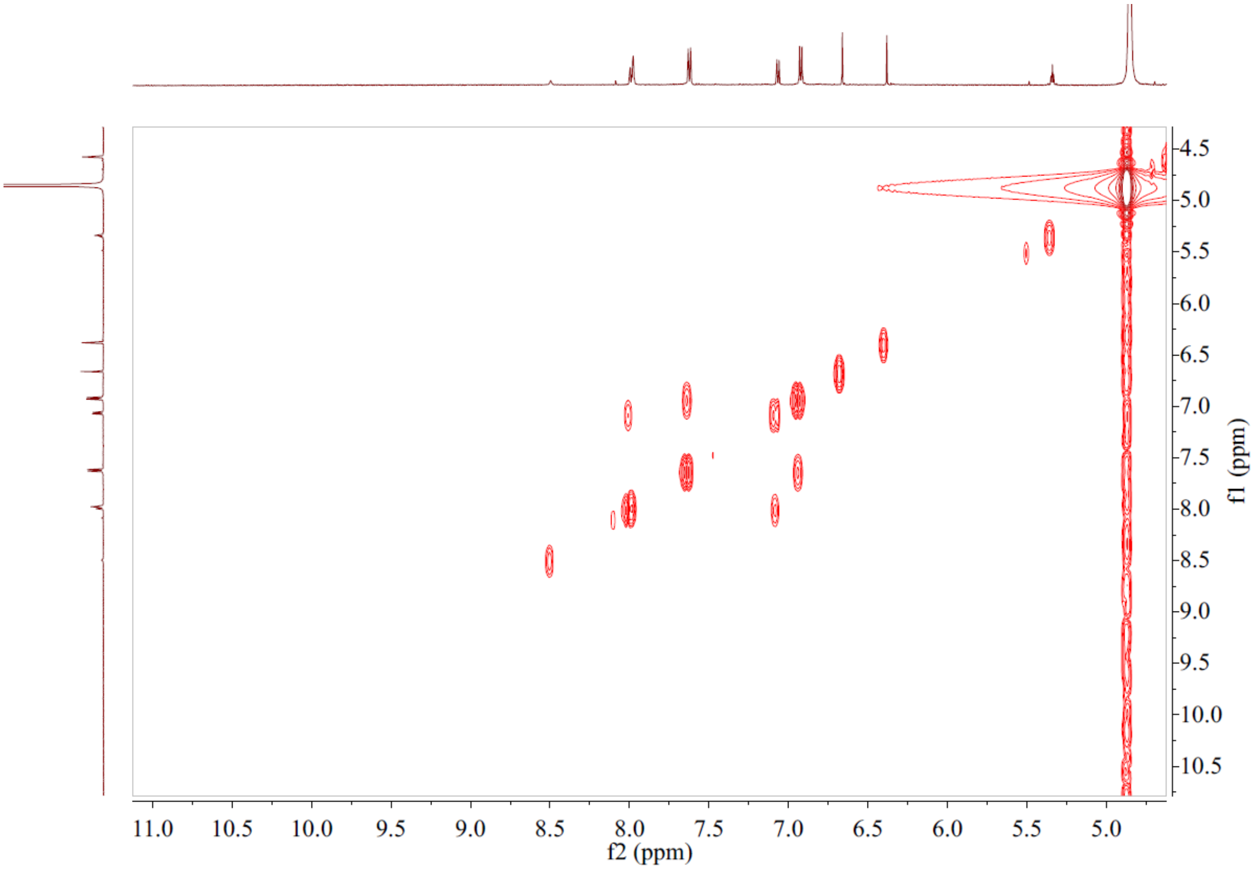

Figure S15. ^{1}H-^{1}H COSY (600 MHz) spectrum of compound **3** in Methanol-*d*4

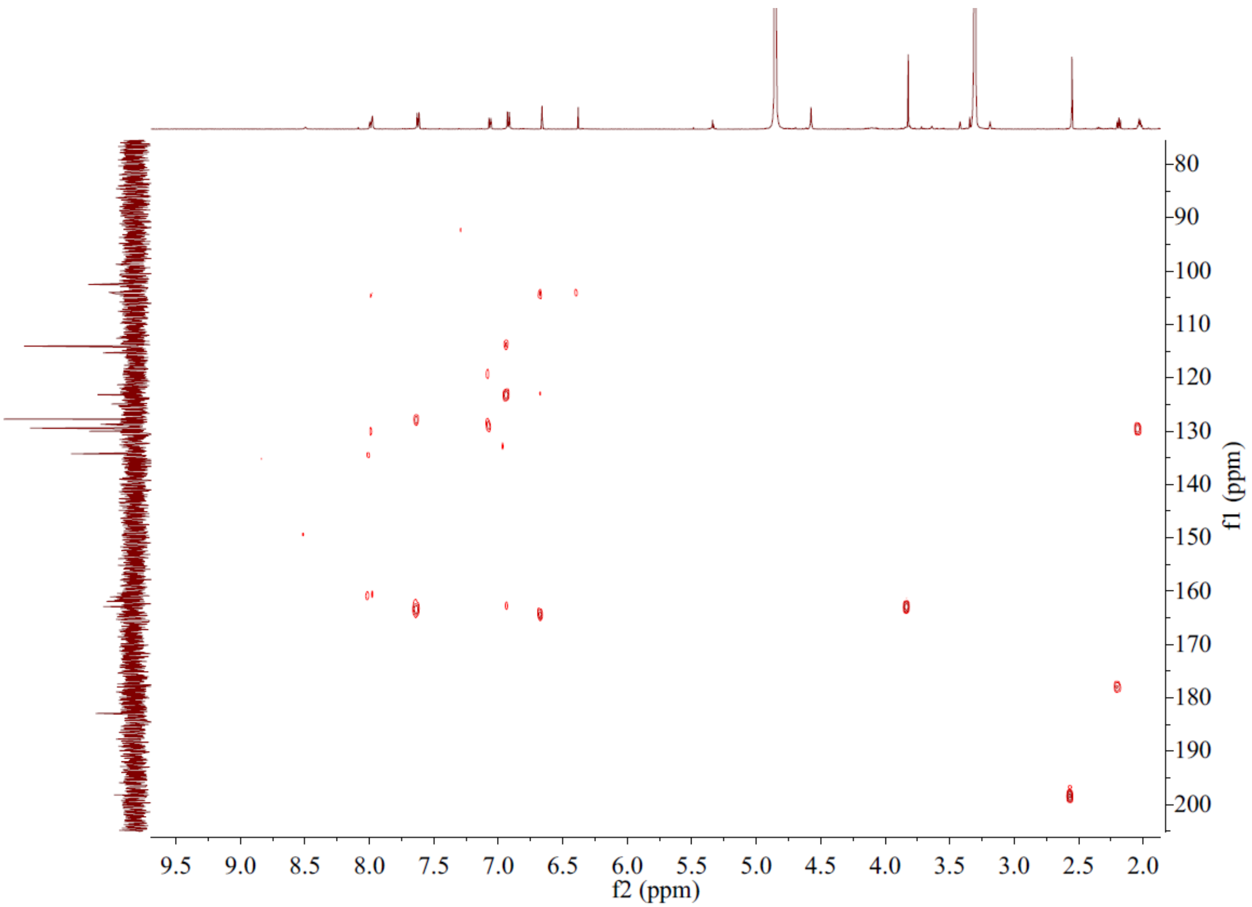

Figure S16. HMBC (600 MHz) spectrum of compound **3** in Methanol-*d*4

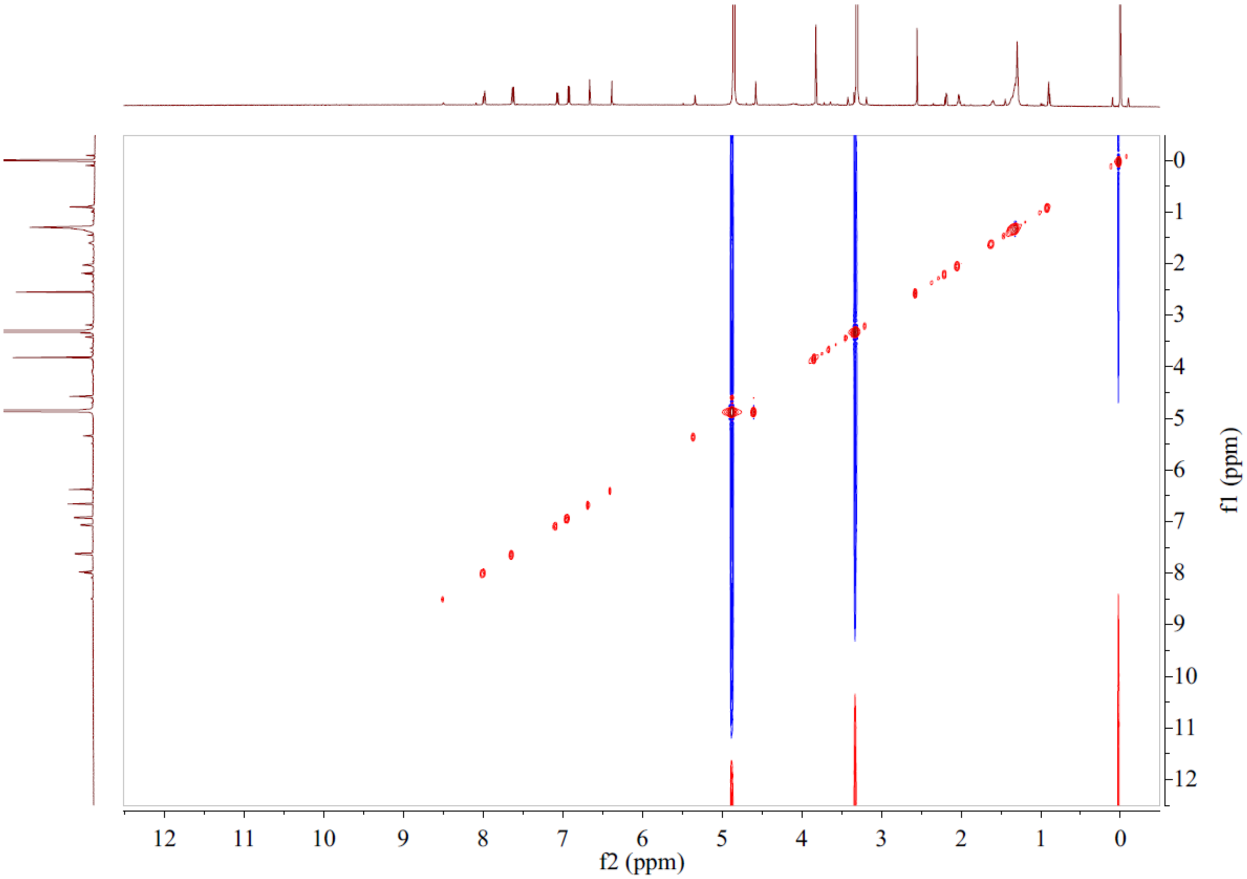

Figure S17. NOESY (600 MHz) spectrum of compound **3** in Methanol-*d*4
